# Taxonomy of the Palearctic socially parasitic *Temnothorax* (*Myrmoxenus*) ants (Hymenoptera: Formicidae)

**DOI:** 10.1371/journal.pone.0308712

**Published:** 2024-10-18

**Authors:** Ferenc Báthori, Bernhard Seifert, Jürgen Heinze, Kadri Kiran, Celal Karaman, Sándor Csősz

**Affiliations:** 1 Department of Systematic Zoology and Ecology, Institute of Biology, ELTE-Eötvös Loránd University, Budapest, Hungary; 2 Department of Entomology, Senckenberg Museum of Natural History Görlitz, Görlitz, Germany; 3 Zoology/Evolutionary Biology, University of Regensburg, Regensburg, Germany; 4 Department of Biology, Trakya University Faculty of Science, Edirne, Türkiye; 5 HUN-REN-ELTE-MTM Integrative Ecology Research Group, Budapest, Hungary; Nanjing Agricultural University, CHINA

## Abstract

The ant genus *Temnothorax* is one of the most diverse in the Palearctic region, comprising several species with different life histories and uncertain taxonomic backgrounds. Socially parasitic *Temnothorax* ant species were typically described decades ago, primarily based on traditional morphological traits. In some aspects, these species have come back into the spotlight in recent years, necessitating a comprehensive taxonomic revision of the species of the genus. In this paper, we present a quantitative morphology-based taxonomic revision of the *Temnothorax corsicus* species group (formerly called *Myrmoxenus* genus) based on the analysis of 20 continuous morphometric traits collected from 394 worker and 19 traits from 473 gyne individuals belonging to 240 samples. Based on morphometric analyses, we propose junior synonymy for *Temnothorax tamarae* (Arnol’di, 1968) under *T*. *ravouxi* (André, 1896), and *T*. *microcellatus* (Soudek, 1925) is revived and is considered a senior synonym of *T*. *menozzii* (Finzi, 1924). Detailed descriptions, measurements, distribution, and host usage of all ten species are given. Dichotomous keys to workers, known gynes, and photographs of all species are presented.

## Introduction

The genus *Temnothorax* Mayr, 1861 is considered one of the most diverse ant genera in the family Formicidae, currently comprising 465 valid species and 35 subspecies [1]. Due to this high number of species and the fact that they are found in a wide variety of environments, from hot semi-desert areas to cold high mountain habitats [2], they are pretty diverse, with a wide range of morphological and behavioral characteristics [3, 4]. These features have previously been the basis of many crucial studies in the fields of behavioral biology [5–8], evolutionary biology [9], taxonomy [10–13] and host-parasite interactions [14–16], making them one of the most studied ants in Europe.

Socially parasitic species of *Temnothorax* occurring in the Palearctic faunal region were previously classified in the genera *Epimyrma* Emery, 1915, *Myrmoxenus* Ruzsky, 1902 *Myrmetaerus* Soudek, 1925, *Chalepoxenus* Menozzi, 1923, *Leptothorax* Mayr, 1855 and *Gonepimyrma* Bernard, 1948 [17–22]. These taxa have undergone a number of taxonomic classification changes over the decades [23–26], but despite different opinions [see Heinze et al. [27]; Seifert et al. [28]], these are all considered synonyms of the currently valid taxa *Temnothorax* [29].

Among the parasitic *Temnothorax* species previously classified in the genera *Myrmoxenus*, twelve of them have long been known to occur in the Palearctic region [16, 27, 30], showing considerable variation in life history. In these species, an evolutionary transition from slave-making to a completely workerless parasitic state can be observed [31]. These species can be active slave-makers with their worker caste, which periodically initiate raiding campaigns [*Temnothorax algerianus* (Cagniant, 1968), *T*. *bernardi* (Espadaler, 1982), *T*. *gordiagini* (Ruzsky, 1902), *T*. *microcellatus* (Soudek, 1925), *T*. *ravouxi* (André, 1896), *T*. *stumperi* (Kutter, 1950)], "degenerate slave-makers" an intermediate step in the loss of the worker caste such as *T*. *kraussei* (Emery, 1916), or true workerless parasitic species [*T*. *adlerzi* (Douwes et al., 1988), *T*. *birgitae* (Schulz, 1994), *T*. *corsicus* (Emery, 1895)]. Most of these species have monogynous colonies, except for *T*. *algerianus*, which has polygynous colonies in some habitats [16, 27, 30, 31]. Only a few species perform mating flights (*T*. *microcellatus*, *T*. *stumperi*, *T*. *ravouxi*), most of them mate within the natal nests (*T*. *adlerzi*, *T*. *algerianus*, *T*. *bernardi*, *T*. *corsicus*, *T*. *kraussei*) [31].

While considering life history traits, a vast majority of these species were described decades ago, often with subjective assessment of individual morphological characters. Kutter pointed out that, in 1973, there was a high degree of intraspecific morphological variation in species of the genus, which is one of the most striking in pedicel [32]. Nevertheless, like their predecessors, later authors considered these morphological characters with doubtful reliability when describing a new species [33]. Despite the dynamic changes in the taxonomy of *Temnothorax* ants today [12, 13, 34], the taxonomy of socially parasitic *Temnothorax* (*Myrmoxenus*) ants of the Palearctic region is relatively untouched, apart from a few studies using modern methods, including phylogenetic analysis [16, 27, 35, 36]. Considering that the description of these species has been done predominantly with traditional taxonomic methods in the last century, a comprehensive taxonomic revision of these parasitic species based on numerical morphology has become quite justified, as already suggested by Seifert [37].

In the present study, we examined more than 800 individuals, workers, and gynes in an integrative taxonomic way using Numeric Morphology Based Alpha Taxonomy (NUMOBAT) and the exploratory data analysis tool NC clustering. Our results will contribute to a better understanding of the taxonomy, biogeography, and host usage of these enigmatic parasitic species in the Palearctic region.

## Materials and methods

### Examined material

The specimens examined belonged to the following institutions: MSNG (Museo Civico di Storia Naturale “Giacomo Doria”, Genova, Italy), NHMB (Naturhistorisches Museum, Basel, Switzerland), MZLU (Zoologiska Museet, Lunds Universitet, Lund, Sweden), NHMP (Natural History Museum, Národní Muzeum, Prague, Czech Republic), MCZC (Museum of Comparative Zoology, Harvard University, Cambridge, Massachusetts, U.S.A.), SMNG (Senckenberg Museum für Naturkunde Görlitz, Görlitz, Germany), MZL (Musée de Zoologie, Lausanne, Switzerland), MNHN (Musee National d’Histoire Naturelle, Paris, France), ZMUM (Zoological Museum of the Moscow State University, Moscow, Russia), SCPC (Private collection of SC) and EMTU (Entomological Museum of Trakya University, Türkiye). Details of the complete morphologically analyzed material (sample codes, collection information, and depository) can be found in S1 Table and the host usage data in S2 Table.

### Morphometric character recording

**Fig 1:**
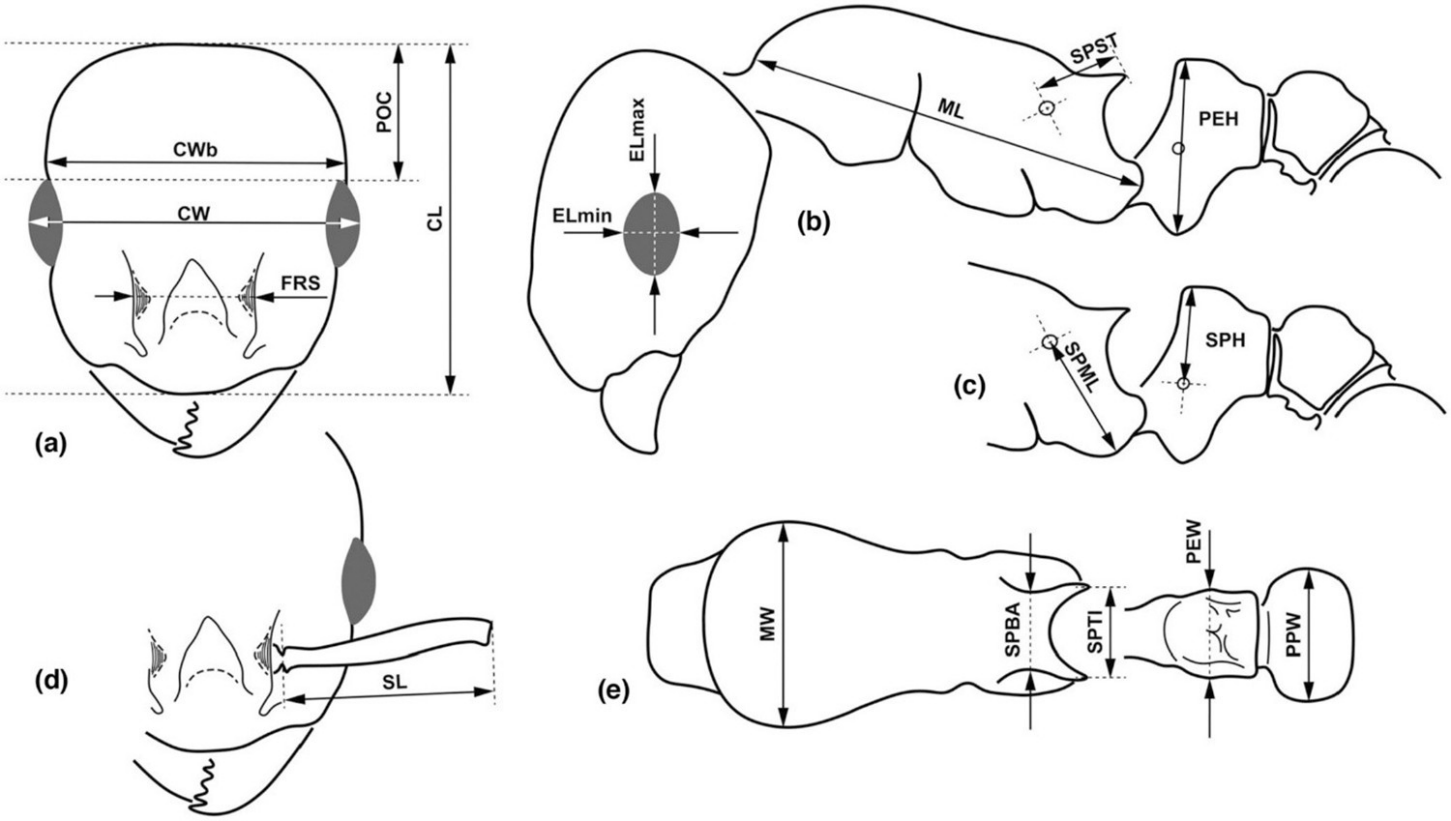
Illustrations for morphometric characters. Head in dorsal view (a) with measurement lines for CL, CW, CWb, FRS and POC; lateral view of mesosoma and head (b) with measurement lines for ELmax, ELmin, ML, PEH and SPST; lateral view of propodeum, petiole and postpetiole (c) with measurement lines for SPML and SPH; frontal region of the head dorsum (d) with measurement lines for SL; dorsal view of mesosoma, petiole and postpetiole (e) with measurement lines for MW, PEW, PPW, SPBA and SPTI.

In the present study, we recorded 20 continuous morphometric traits in 394 workers and 19 traits in 473 gynes from a total of 240 nest samples. All measurements were made via a graticule in μm using a pin-holding stage, permitting rotations around the X, Y, and Z axes. An Olympus SZX16 stereomicroscope was used at a magnification of ×80 (for larger body parts) and ×160 (for smaller traits). All specimens (except two syntypes *Epimyrma algeriana* worker and one paratype *E*. *bernardi* worker measured by Henri Cagniant) were measured by FB, and one type specimen could only be measured by software (by FB) from images, as the Zoological Museum of the Moscow State University does not currently send out specimens. Definitions of morphometric characters are detailed in Table 1 and Fig 1. The longest setae of the petiole (HPL) and Pronotum (PnHL) were measured in three species (*T*. *ravouxi*, *T*. *algerianus*, *T*. *kraussei*). The individuals examined were randomly selected by generating random numbers for the individuals available to us using the "random" function in Microsoft Excel. Then, after ascending order, the first twenty workers and gynes were examined for all three species. Raw data in millimeters are given in S1 Table.

**Table 1:** Verbatim trait definitions for morphometric character recording.

Abbr.	Verbal definition of the trait	See:
**CL**:	maximum cephalic length in median line; the head must be carefully tilted to the position with the true maximum. Excavations of hind vertex and/or clypeus, if any, reduce CL	(Fig 1A).
**CS**:	cephalic size; the arithmetic mean of CL and CWb	
**CW**:	maximum width of the head capsule, measured across the eyes	(Fig 1A).
**CWb**:	maximum width of the head capsule, measured posterior to the eyes	(Fig 1A).
**ELmax**:	maximum diameter of the compound eye	(Fig 1B).
**ELmin**:	minimum diameter of the compound eye	(Fig 1B).
**FRS**:	minimum distance between the frontal carinae	(Fig 1A).
**ML**:	mesosoma length from caudalmost point of the propodeal lobe to the transition point between anterior pronotal slope and anterior propodeal shield (preferentially measured in lateral view; if the transition point is not well defined, use dorsal view and take the center of the dark-shaded borderline between pronotal slope and pronotal shield as an anterior reference point)	(Fig 1B).
**MW**:	maximum mesosoma width; pronotal width in workers	(Fig 1E).
**PEH**:	maximum petiole height.	(Fig 1B).
**PEW**:	maximum width of petiole	(Fig 1E).
**PHL**	length of longest hair on petiole	
**PnHL**	length of the longest hair on pronotum	
**POC**:	postocular distance. Use a cross-scaled ocular micrometer and adjust the head to the measuring position of CL. Caudal measuring point: median occipital margin; frontal measuring point: median head at the level of the posterior eye margin	(Fig 1A).
**PPH**	maximum height of postpetiole	
**PPW**:	maximum width of postpetiole	(Fig 1E).
**SL**:	maximum straight line scape length excluding the articular condyle.	(Fig 1D)
**SPBA**:	the smallest distance between the lateral margins of the spines at their base. This should be measured in the dorsofrontal view since the broader parts of the ventral propodeum do not interfere with the measurement in this position. If the lateral margins of spines diverge continuously from the tip to the base, the smallest distance at the base is not defined. In this case, SPBA is measured at the level of the bottom of the interspinal meniscus	(Fig 1E).
**SPH**:	maximum height of the petiolar node, measured in lateral view from the petiolar spiracle	(Fig 1C)
**SPML**	distance between the center of the propodeal stigma and the metapleural margin	(Fig 1C)
**SPST**:	distance between the center of the propodeal stigma and the spine tip. The stigma center refers to the midpoint defined by the outer cuticular ring but not to the center of the real stigma opening that may be positioned eccentrically	(Fig 1B).
**SPTI**:	the distance between spine tips in dorsal view; if spine tips are rounded or truncated, the centers of spine tips are taken as reference points	(Fig 1E).
**SPWI**:	maximum distance between outer margins of spines, measured in the same position as SPBA.	(Fig 1E)

Abbreviations and definitions of measured morphological characters.

### Multivariate morphological analyses

#### Exploratory analyses via NC-PART clustering

The prior species hypothesis was generated based on workers via a combined application of NC clustering [38] and Partitioning Based on Recursive Thresholding (PART) [39]. The protocol was published by Csősz & Fisher [40], which is now applied with the following specific setups: bootstrap iterations in PART were set to ’b = 1000’, and the minimum size of clusters was set to ’minSize = 5’ for both ‘hclust’ and ‘means.’ The optimal number of clusters and the Partitioning of samples are accepted as preliminary species hypotheses in every case when the two clustering methods, ’hclust’ and ‘kmeans’ via PART, have arrived at the same conclusion.

#### Exploratory analyses via PCA using allometrically corrected data

An alternative prior species hypothesis has been generated via the ordinating Principal Component Analysis (PCA), which displays plots in a graphic. Allometries were calculated via regression analyses for every trait on CL as the independent variable, and residuals were applied.

#### Hypothesis testing by confirmatory analyses

The validity of the prior morphospecies hypothesis was tested by LDA, in which scatterplots and a histogram illustrate the distribution of the individuals in a morphospace. Leave-one-out cross-validation LDA (LOOCV-LDA) returns a more conservative estimate of the validity of the particular model based on repeating the leave-one-out process for every data point against the model and testing its predicted position.

#### Imaging

Z-stack images of mounted ants were produced with Keyence, a VHX 7000 digital microscope, using the multi-lightning mode at magnifications between 80× and 150×. Figures created using the free PhotoScape v3.7 software and Paint 11.2402.32.0.

#### Distribution maps

The distribution map based on the studied samples was created using QGIS software version 3.10.6 [41].

## Results and discussion

Altogether, ten species are recognized through morphometric analyses.

**Fig 2:**
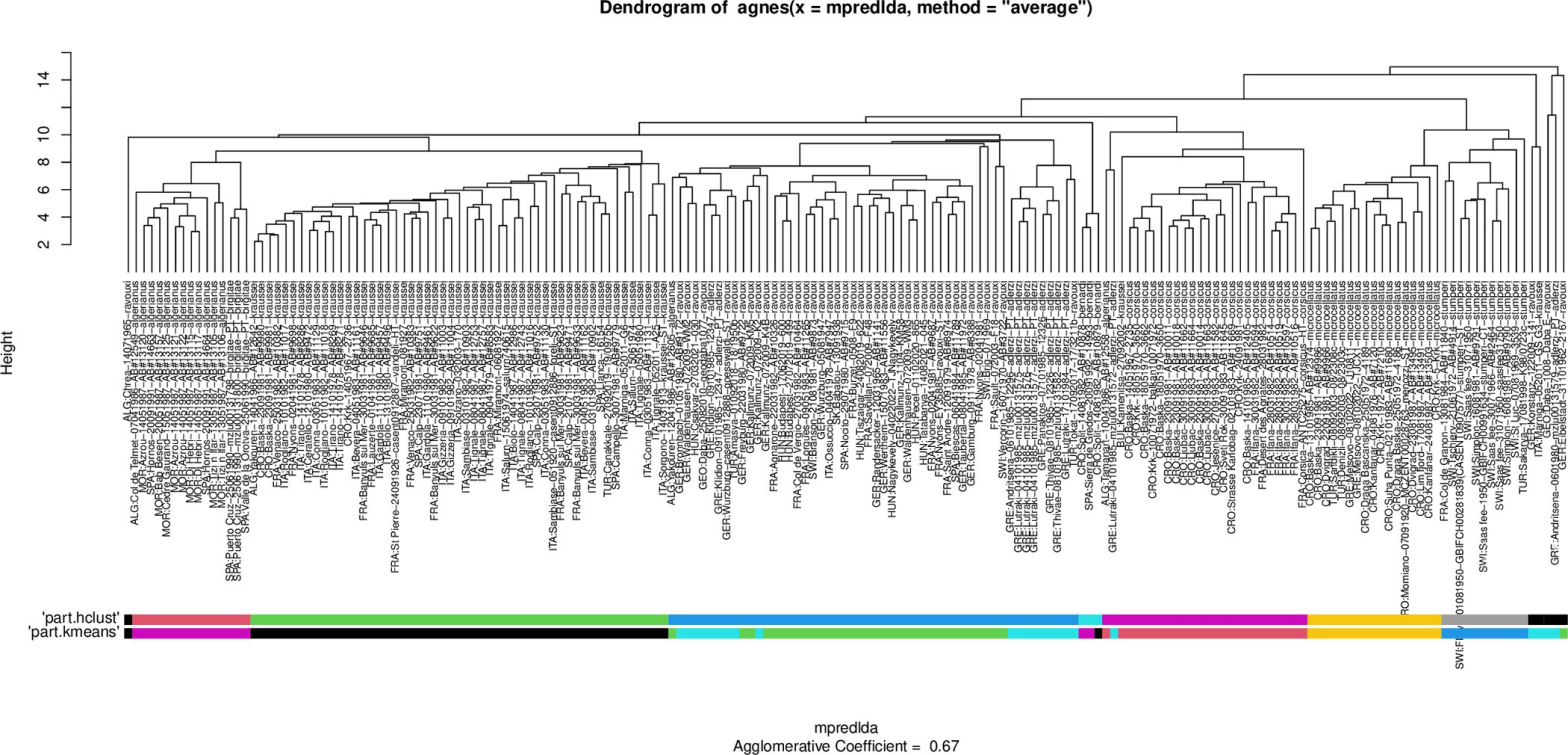
Dendrogram solution for morphometric data of *Temnothorax corsicus* group species [29] in NC clustering using the UPGMA distance method. The pattern is calculated from raw data of gyne specimens; the labels represent nest samples. Bars represent "kmeans" and "hclust” partitioning results returned by function PART.

**Fig 3:**
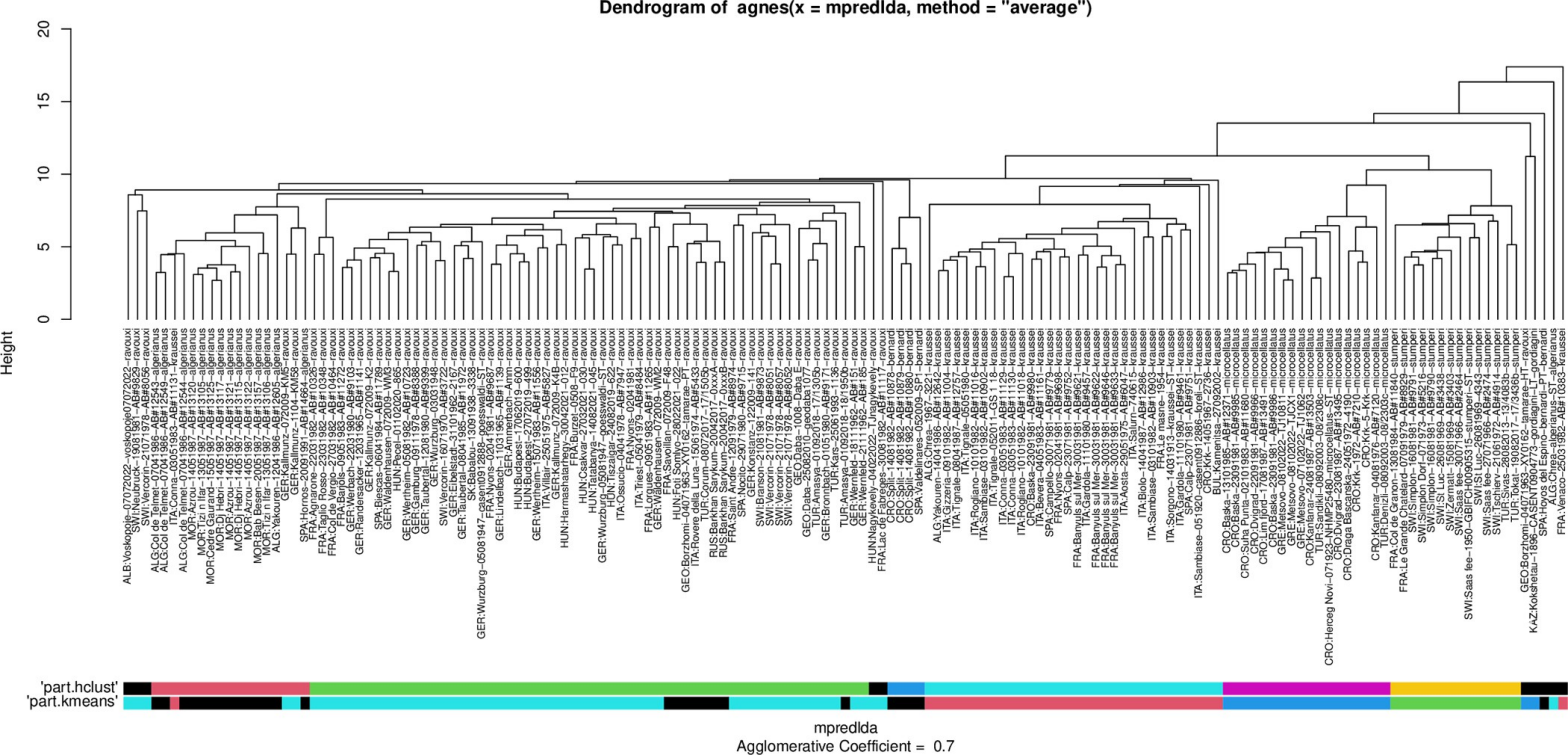
Dendrogram solution for morphometric data of *Temnothorax corsicus* group species in NC clustering using UPGMA distance method. The pattern is calculated from raw data of worker specimens, the labels represent nest samples. Bars represent “kmeans”, and “hclust” partitioning results returned by function PART.

In gynes (female sexuals), eight clusters were identified by ‘means’ clustering algorithms and seven in ‘hclust’ and using the function ’part.’ The pattern recognized by these partitioning algorithms can be fitted on the hierarchical structure seen on the dendrogram generated by NC clustering (Fig 2). Note that the small sample size (three nest samples) hampered recognition of the Canarian endemic species *T*. *birgitae*, because the minimum recognizable cluster size was set to n = 5, and the *T*. *gordiagini* gyne is unknown, only the holotype worker is available. As the paratype gyne of *T*. *tamarae* clustered with *T*. *ravouxi* together with other *T*. *tamarae* specimens collected from Georgia near the type locality (Borzhomi and Daba) (Figs 2 and 3), we will henceforth treat this species as a synonym of *T*. *ravouxi* (see below).

The grouping hypotheses generated by the combination of hypothesis-free exploratory analyses were validated by linear discriminant analysis with leave-one-out cross-validation (LOOCV-LDA). The overall classification success in gynes is 97%, and the overall accuracy is 0.97 [95% CI: (0.95, 0.99)] (Table 2).

**Table 2:** Cross validation LDA classification table for gyne individuals.

	*adlerzi*	*algerianus*	*bernardi*	*birgitae*	*corsicus*	*kraussei*	*microcellatus*	*ravouxi*	*stumperi*
***adlerzi***	13	0	0	0	0	0	0	3	0
***algerianus***	0	31	0	0	0	1	0	0	0
***bernardi***	0	0	7	0	0	0	0	0	0
***birgitae***	0	0	0	8	0	0	0	0	0
***corsicus***	0	1	0	0	74	1	0	0	0
***kraussei***	0	0	0	0	0	90	0	1	0
***microcellatus***	0	0	0	0	0	0	30	0	0
***ravouxi***	2	2	0	0	0	0	0	98	0
***stumperi***	0	0	0	0	0	0	0	0	18

In workers six clusters were identified by both ‘kmeans’ and ‘hclust’ clustering algorithms using function ‘part’ due to three species (*T*. *adlerzi*, *T*. *birgitae*, *T*. *corsicus*) are workerless. The pattern recognized by these partitioning algorithms can be fitted on the hierarchical structure seen on the dendrogram generated by NC clustering (Fig 3).

The grouping hypotheses generated by the hypothesis-free NC clustering analyses was validated by Linear Discriminant Analysis with leave-one-out cross-validation (LOOCV-LDA). The overall classification success in workers is 97%, overall accuracy: 0.97 [95% CI: (0.95, 0.99)] (Table 3).

**Table 3:** Cross validation LDA classification table for worker individuals. The only specimen of *T*. *gordiagini* was not analyzed.

	*algerianus*	*bernardi*	*kraussei*	*microcellatus*	*ravouxi*	*stumperi*
***algerianus***	41	0	1	1	2	0
***bernardi***	1	9	1	0	0	0
***kraussei***	1	0	58	0	1	0
***microcellatus***	0	0	0	39	1	0
***ravouxi***	2	2	0	0	156	0
***stumperi***	0	0	0	0	1	32

The phenotipically distinguishable clusters represent nine morphologically diagnosable OTUs, which is made complete with the *T*. *gordiagini*, which is represented by the holotype only. Hence the ten-species solution is accepted as the final species hypothesis. These species differ in quantitative traits (see Table 1) and many qualitative characters (e.g. shape of propodeal spines, petiolar node, surface sculpturing etc.).

The systematic position of *Epimyrma* (*Gonepimyrma*) *africana* Bernard, 1948.

**Fig 4:**
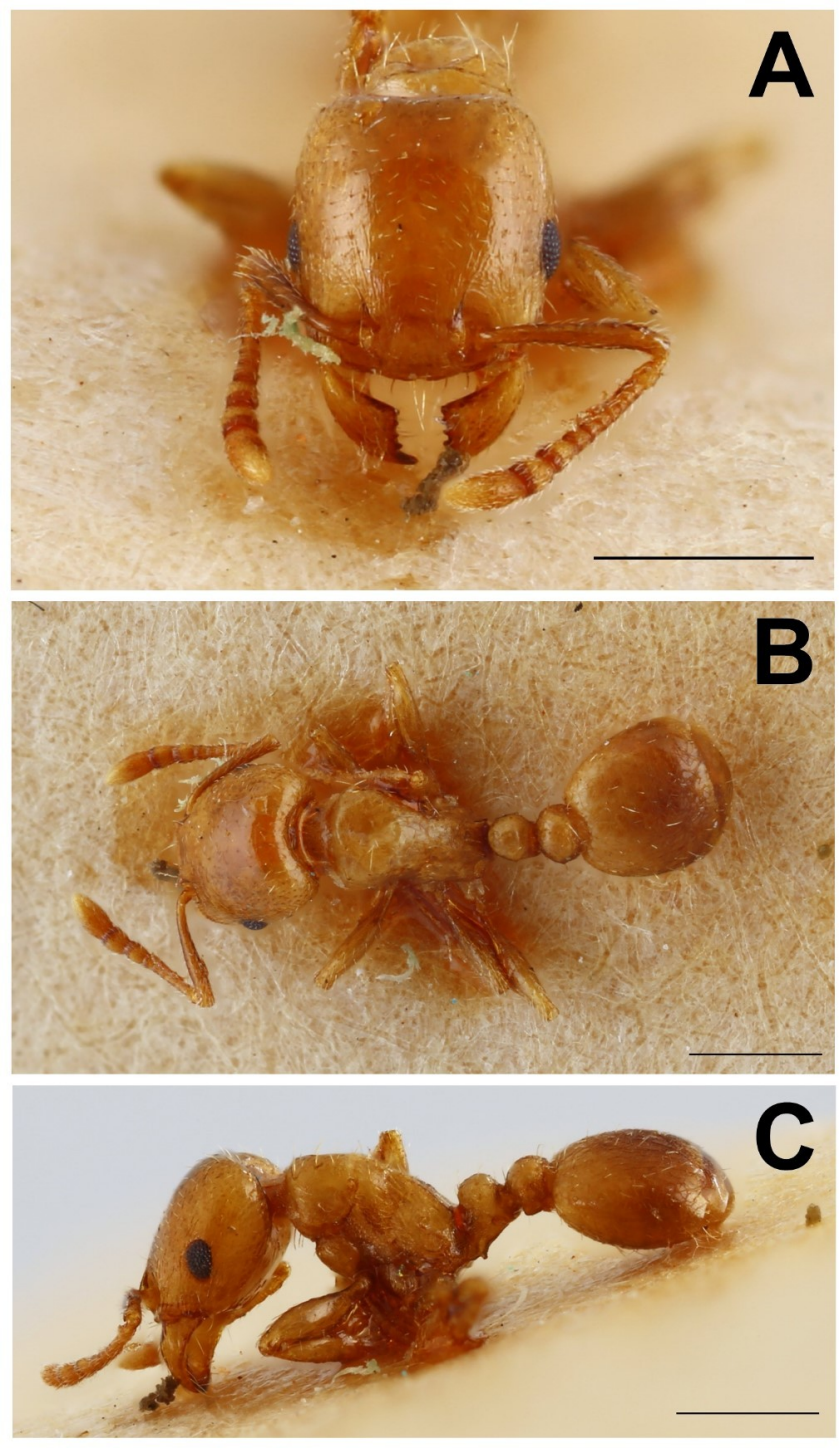
*Epimyrma (Gonepimyrma) africana* Bernard, 1948 holotype worker (MNHN collection number: EY36212). Head in full-face view (A), dorsal view of the body (B), lateral view of the body (C), scale bar: 0.5 mm.

**Fig 5:**
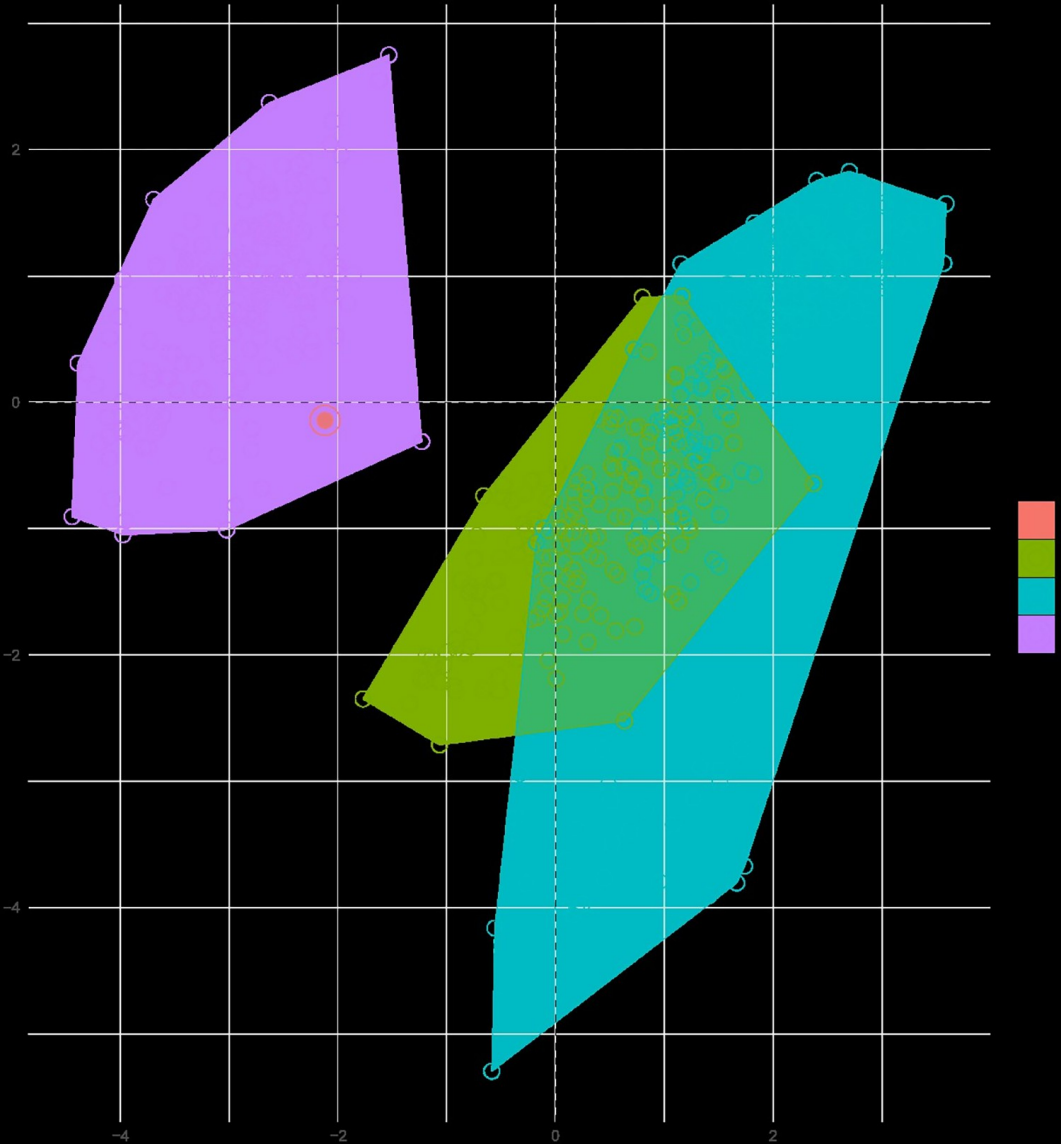
PCA plot of morphometric data of *Temnothorax corsicus* group species (green), *Nesomyrmex angulatus* group species (blue), and *Tetramorium* (lillac) nest samples and type material of *Tetramorium epimyrmoide* [formerly *Epimyrma* (*Gonepimyrma*) *africana* Bernard, 1948] (red) illustrated on two principal components (Dim 1, Dim 2).

Although the accuracy of the classification has been questioned, this taxon has been reported by several researchers [31, 33, 42, 43] as an *Epimyrma* species, *E*. *africana* (Fig 4), and was reclassified as *Temnothorax* by Ward et al. [28]. Despite the fact that although he was unable to examine the type specimen personally, Kutter [32] questioned the species classification and suggested from the original description that the specimen might actually belong to *Leptothorax* (*Goniothorax) angulatus*. However, investigation of the holotype worker suggests that this taxon is not a *Temnothorax* species but belongs to *Tetramorium* and is most likely a member of the *Tetramorium taueret* or *T*. *sahlbergi* complex. The PCA analyses of this sample confirmed this view. The *Temnothorax africanus* holotype worker is nested in the *Tetramorium* cluster in a morphospace generated based on morphometric data of 198 *Temnothorax* (*Myrmoxenus*), 266 *Nesomyrmex angulatus*-group species and 431 *Tetramorium* nest samples (Fig 5). Therefore, we propose its placement in the genus *Tetramorium*. However, since *Tetramorium africanum* (Mayr, 1866) is already described, we propose a replacement name *Tetramorium epimyrmoide*, for this taxon.

Synopsis of *Temnothorax corsicus* species group

*Temnothorax adlerzi* (Douwes et al., 1988)

*Temnothorax algerianus* (Cagniant, 1968)

*Temnothorax birgitae* (Schulz, 1994)

*Temnothorax bernardi* (Espadaler, 1982)

*Temnothorax corsicus* (Emery, 1895)

*Temnothorax gordiagini* (Ruzsky, 1902)

*Temnothorax kraussei* (Emery, 1915)

*Temnothorax vandeli* (Santschi, 1927): Buschinger et al. [44]: 274

*Temnothorax foreli* Menozzi, 1921: Buschinger et al. [44]: 274

*Temnothorax microcellatus* (Soudek, 1925) **revived from synonymy**

*menozzii* syn. nov.

*Temnothorax ravouxi* (André, 1896)

*Temnothorax goesswaldi*: Buschinger [45]: 352

*Temnothorax tamarae* (Arnol’di, 1968) **syn. n.**

*Temnothorax zaleskyi*: Báthori et al. [16]: 4.

*Temnothorax stumperi* (Kutter, 1950)

Key to gynes

1a Endemic to the Canary Islands…***birgitae***
1b Out of Canary Island, West Palearctic…2
2a Funiculus 11 segmented…3
2b Funiculus 10 segmented…4
3 Pontic-mediterranean species…***microcellatus***

This species is known from its type locality only: Kazakhstan. Gyne unknown…***gordiagini***

4a Anterior margin of clypeus flat…***stumperi***
4b Anterior margin of clypeus evenly convex…5
5a Head frons shiny, ground sculpture absent or feeble areolate…6
5b Head frons dull, ground sculpture areolate…7
6a Head frons extensively smooth with very feeble or absent sculpture…***bernardi***
6b Head frons medially shiny, feebly costulate laterally…***corsicus***
7a Longest erect hair of the petiole >140μm…***kraussei***
7b Longest erect hair of the petiole <140μm…8
8a North Africa, South Spain…***algerianus***
8b North Spain to Türkiye…***adlerzi***, ***ravouxi*** [for safe separation see differential diagnosis under *T*. *adlerzi*]

Key to workers

1a Funiculus 11 segmented…2
1b Funiculus 10 segmented…3
2a In profile, the dorsal contour line of the propodeum is straight…***gordiagini***
2b In profile, the dorsal contour line of the propodeum is convex…***microcellatus***
3a Anterior margin of clypeus straight…***stumperi***
3b Anterior margin of clypeus convex…4
4a Head frons shiny, ground sculpture absent or feebly areolate …***bernardi***
4b Head frons dull, ground sculpture areolate …5
5a Longest erect hair on petiole >125μm…***kraussei***
5b Longest erect hair on petiole <125μm…6
6a North Africa, South Spain…***algerianus***
6b North Spain to Türkiye…***ravouxi***

### *Temnothorax adlerzi* (Douwes et al., 1988)

*Epimyrma adlerzi* Douwes et al. [33]: 240 (q.m.) GREECE.

Combination in *Myrmoxenus*: Schulz & Sanetra [25]: 167.

Combination in *Temnothorax*: Ward et al. [29]: 75.

Type material investigated.

11 Paratype gynes were investigated from the type localities: Greece: Blue Lake W Loutraki, 17.X.1984, leg. Douwes, Jessen & Buschinger (1g-ZMLS, 1g-NHMB); Greece: Blue Lake W Loutraki, 4.X.1985, leg. Buschinger-Douwes-Heinze-Jessen-Winter (4g-ZMLS); Greece: S Andritsena, Bassai, 6.x.1985, leg. Buschinger-Douwes-Heinze-Jessen-Winter (1g-ZMLS, 1g-NHMB); Greece: Thivae, Kaza, 8.X.1985, leg. Buschinger-Douwes-Heinze-Jessen-Winter (1g-ZMLS, 1g-NHMB); Greece: Florina, Klidion, 9.X.1985 (1g-NHMB), leg. Buschinger, Douwes, Heinze, Jessen, Winter.

**Fig 6:**
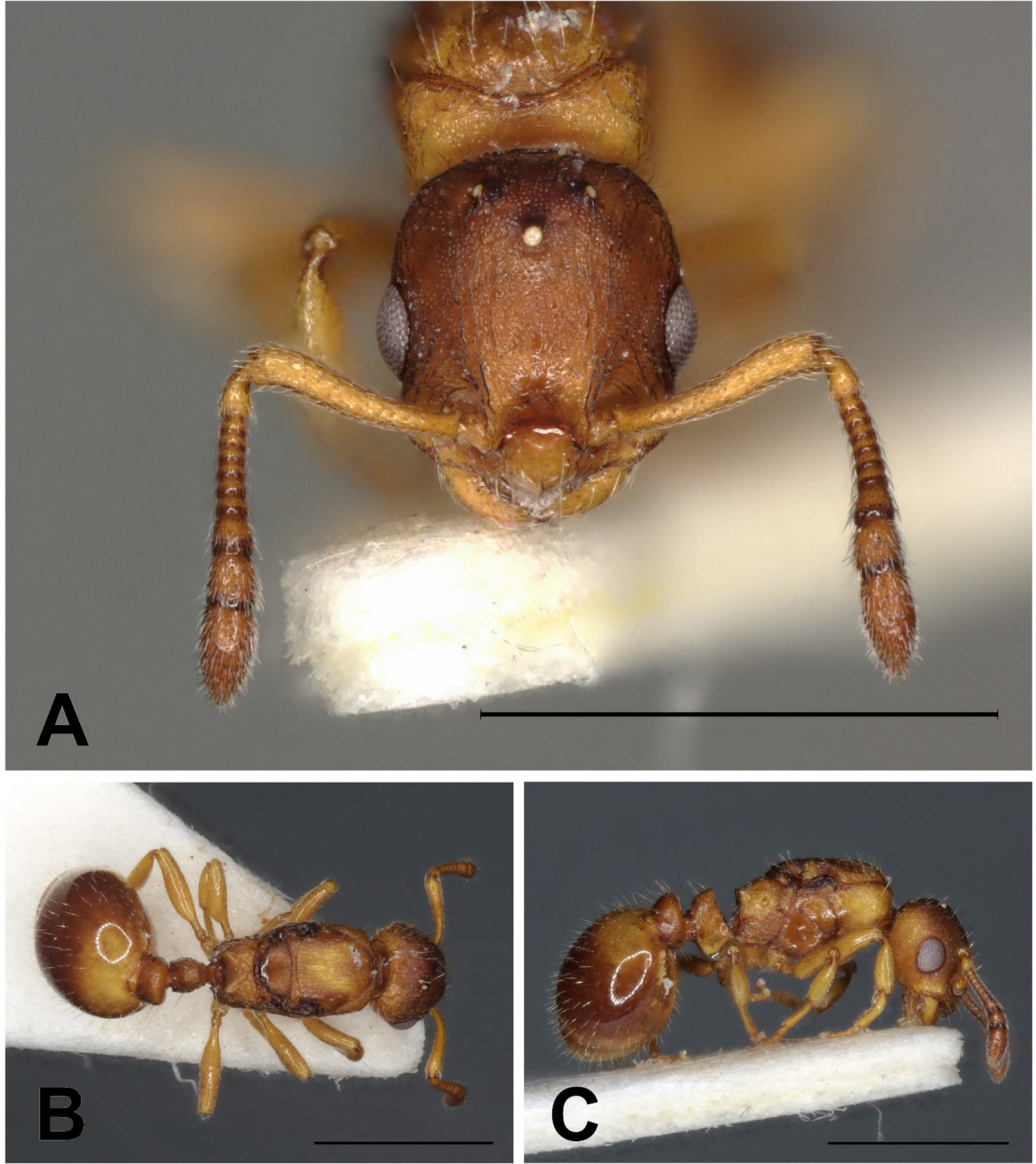
*Temnothorax adlerzi* gyne. Head in full-face view (A), dorsal view of the body (B), lateral view of the body (C), scale bar: 1 mm.

Description of gynes. (Fig 6 and Table 4).

**Table 4:** Mean of morphometric ratios calculated based on gyne individuals.

	*adlerzi* (n = 17)	*algerianus* (n = 34)	*bernardi* (n = 7)	*birgitae* (n = 8)	*corsicus* (n = 92)	*kraussei* (n = 124)	*microcellatus* (n = 50)	*ravouxi* (n = 121)	*stumperi* (n = 20)
**CS**	621±21.665	615±18.398	589±10.618	589±24.456	593±18.745	624±21.793	605±15.596	635±20.777	583±8.563
** **	[571, 653]	[552, 649]	[576, 609]	[554, 634]	[544, 640]	[569, 674]	[569, 639]	[591, 675]	[569, 604]
**CL/CWb**	1.181±0.014	1.184±0.019	1.186±0.013	1.161±0.020	1.207±0.024	1.213±0.024	1.127±0.021	1.181±0.025	1.097±0.024
** **	[1.149, 1.205]	[1.151, 1.225]	[1.163, 1.205]	[1.135, 1.192]	[1.129, 1.266]	[1.148, 1.280]	[1.080, 1.198]	[1.135, 1.250]	[1.061, 1.148]
**POC/CL**	0.364±0.009	0.373±0.007	0.348±0.005	0.376±0.014	0.356±0.012	0.353±0.008	0.365±0.009	0.362±0.011	0.363±0.009
** **	[0.335, 0.338]	[0.356, 0.390]	[0.341, 0.355]	[0.363, 0.400]	[0.320, 0.384]	[0.332, 0.375]	[0.348, 0.384]	[0.327, 0.411]	[0.350, 0.382]
**SL/CL**	0.650±0.029	0.681±0.014	0.694±0.012	0.718±0.013	0.625±0.017	0.687±0.019	0.714±0.019	0.651±0.017	0.718±0.019
** **	[0.555, 0.567]	[0.649, 0.702]	[0.680, 0.708]	[0.702, 0.738]	[0.575, 0.658]	[0.635, 0.733]	[0.666, 0.761]	[0.610, 0.699]	[0.677, 0.757]
**FR/CS**	0.330±0.011	0.357±0.013	0.366±0.007	0.341±0.016	0.364±0.009	0.336±0.010	0.353±0.009	0.357±0.015	0.343±0.012
** **	[0.331, 0.334]	[0.328, 0.380]	[0.351, 0.372]	[0.305, 0.356]	[0.340, 0.388]	[0.307, 0.365]	[0.335, 0.370]	[0.324, 0.395]	[0.320, 0.359]
**MW/CS**	0.864±0.027	0.842±0.029	0.878±0.033	0.820±0.026	0.796±0.038	0.809±0.028	0.853±0.042	0.890±0.030	0.850±0.037
** **	[0.882, 0.892]	[0.795, 0942]	[0.850, 0.944]	[0.791, 0.873]	[0.711, 0.886]	[0.711, 0.893]	[0.781, 0.946]	[0.806, 0.967]	[0.795, 0.925]
**SPWI/CS**	0.383±0.017	0.407±0.015	0.455±0.010	0.406±0.020	0.406±0.019	0.389±0.020	0.378±0.015	0.410±0.020	0.316±0.013
** **	[0.347, 0.422]	[0.374, 0.443]	[0.437, 0.467]	[0.377, 0.437]	[0.345, 0.456]	[0.323, 0.443]	[0.338, 0.421]	[0.361, 0.463]	[0.287, 0.342]
**SPTI/CS**	0.337±0.017	0.368±0.010	0.413±0.007	0.363±0.019	0.366±0.024	0.359±0.019	0.325±0.013	0.368±0.018	0.269±0.014
** **	[0.314, 0.366]	[0.340, 0.383]	[0.400, 0.422]	[0.337, 0.394]	[0.300, 0.414]	[0.281, 0.399]	[0.301, 0.359]	[0.330, 0.409]	[0.237, 0.292]
**PEW/CS**	0.309±0.013	0.299±0.014	0.319±0.012	0.312±0.010	0.312±0.013	0.281±0.018	0.285±0.013	0.304±0.016	0.273±0.009
** **	[0.277, 0.328]	[0.270, 0.333]	[0.301, 0.337]	[0.302, 0.326]	[0.285, 0.346]	[0.245, 0.365]	[0.267, 0.350]	[0.258, 0.339]	[0.254, 0.287]
**PPW/CS**	0.448±0.014	0.427±0.017]	0.496±0.012	0.462±0.011	0.469±0.018	0.432±0.019	0.445±0.020	0.452±0.017	0.418±0.013
** **	[0.423, 0.480]	[0.404, 0.491]	[0.482, 0.511]	[0.447, 0.483]	[0.431, 0.509]	[0.386, 0.509]	[0.409, 0.519]	[0.406, 0.500]	[0.390, 0.436]
**ML/CS**	1.452±0.038	1.458±0.034	1.489±0.014	1.445±0.029	1.379±0.040	1.421±0.039	1.473±0.036	1.517±0.034	1.444±0.166
** **	[1.381, 1.533]	[1.388, 1.503]	[1.474, 1.517]	[1.410, 1.475]	[1.280, 1.465]	[1.162, 1.490]	[1.391, 1.578]	[1.408, 1.589]	[1.396, 1543]
**SPST/CS**	0.284±0.010	0.250±0.013	0.247±0.009	0.263±0.009	0.250±0.013	0.251±0.013	0.249±0.009	0.266±0.013	0.263±0.006
** **	[0.269, 0.300]	[0.209, 0.275]	[0.238, 0.258]	[0.249, 0.275]	[0.207, 0.283]	[0.212, 0.287]	[0.224, 0.264]	[0.230, 0.304]	[0.251, 0.277]
**PLST/CS**	0.395±0.010	0.396±0.011	0.396±0.010	0.405±0.014	0.385±0.016	0.393±0.015	0.418±0.015	0.400±0.020	0.393±0.010
** **	[0.373, 0.412]	[0.373, 0.414]	[0.380, 0.412]	[0.389, 0.435]	[0.337, 0.429]	[0.357, 0.427]	[0.394, 0.462]	[0.329, 0.447]	[0.373, 0.407]
**PEH/CS**	0.622±0.012	0.583±0.017	0.630±0.012	0.591±0.014	0.601±0.023	0.570±0.020	0.582±0.019	0.620±0.026	0.588±0.018
** **	[0.600, 0.639]	[0.538, 0.615]	[0.612, 0.650]	[0.568, 0.605]	[0.532, 0.670]	[0.528, 0.630]	[0.554, 0.626]	[0.551, 0.705]	[0.558, 0.630]
**SPH/CS**	0.288±0.019	0.255±0.018	0.295±0.009	0.296±0.019	0.278±0.018	0.277±0.016	0.328±0.028	0.293±0.020	0.279±0.019
** **	[0.259, 0.329]	[0.222, 0.305]	[0.283, 0.308]	[0.272, 0.329]	[0.239, 0.317]	[0.220, 0.320]	[0.280, 0.389]	[0.215, 0.336]	[0.243, 0.330]
**PPH/CS**	0.491±0.009	0.479±0.020	0.514±0.012	0.485±0.015	0.484±0.015	0.458±0.017	0.433±0.019	0.484±0.022	0.449±0.018
** **	[0.468, 0.505]	[0.431, 0.528]	[0.500, 0.531]	[0.458, 0.504]	[0.452, 0.521]	[0.420, 0.511]	[0.399, 0.476]	[0.438, 0.542]	[0.416, 0.480]
**EYE/CS**	0.296±0.009	0.274±0.007	0.299±0.005	0.274±0.014	0.265±0.009	0.271±0.009	0.313±0.009	0.292±0.009	0.291±0.008
	[0.279, 0.314]	[0.257, 0.291]	[0.290, 0.306]	[0.240, 0.284]	[0.241, 0.285]	[0.216, 0.293]	[0.288, 0.339]	[0.265, 0.311]	[0.280, 0.312]

Morphometric traits are divided by cephalic size (CWb), ±SD are provided in the upper row, minimum and maximum values are given in parentheses in the lower row.

Body color brown. Body color pattern concolorous.

Head.

Absolute cephalic size: 621±21.665 [571, 653]. Cephalic length vs. maximum width of head capsule (CL/CWb): 1.181±0.014 [1.149, 1.205]. Color: Brown. Frons: Dark brown. Vertex: Dark brown. Scape: Pale brown. Antennal club: Dark brown. Head frontal sculpture: rugoso-reticulate with areolate ground sculpture. Moderately shiny. Mandibles: Smooth, 4–5 teeth, basal tooth, t2-t3 short, reducated, apical tooth, t1 long. Clypeus: Smooth with median and lateral ridges, median one not reaching posterior margin. Mandibles: Smooth. Anterior margin of clypeus: Strongly convex, slightly pointed. Hairs: Hairs erect allover except adpressed on the antennae. Postocular distance vs. cephalic length (POC/CL): 0.364±0.009 [0.335, 0.338]. Eye vs. absolute cephalic size (EYE/CS): 0.296±0.009 [0.279, 0.314]. Frontal carina distance vs. absolute cephalic size (FR/CS): 0.330±0.011 [0.331, 0.334]. Scape length vs. absolute cephalic size (SL/CL): 0.650±0.029 [0.555, 0.567].

Alitrunk.

Color and color pattern: Pale brown usually with irregular darker margins laterally and characteristic dark margins along anterior margin of mesonotum and longitudinal spot on each side. Scutellum at least partly dark brown. Sometimes whole alitrunk dark brown with indistinct markings. Sculpture: Dorsal region of mesosoma rugulose with rugoso-areolate ground sculpture, sometimes with parallel costulate main sculpture. Lateral region of pronotum rugoso-areolate. Anepisternum areolate lower third smooth, shiny. Katepisternum smooth, shiny, upper third areolate. Metapleuron rugoso-areolate. Propodeal spines: Very short, dull and triangulate. Hairs: Relatively dense erect hairs especially on pro- and mesonotum and on the posterior face of propodeum. Mesosoma length vs. absolute cephalic size (ML/CS): 1.452±0.038 [1.381, 1.533]. Maximum mesosoma width vs. absolute cephalic size (MW/CS): 0.864±0.027 [0.818, 0.892]. Spine length vs. absolute cephalic size (SPST/CS): 0.284±0.010 [0.269, 0.300]. Minimum spine distance at its base vs. absolute cephalic size (SPBA/CS): NA. Maximum spine distance at its tip vs. absolute cephalic size (SPWI/CS): 0.383±0.017 [0.347, 0.422]. Apical spine distance vs. absolute cephalic size (SPTI/CS): 0.337±0.017 [0.314, 0.366].

Pedicel.

Color: Brown, pale brown. Sculpture: Petiole and postpetiole nodes faintly punctuated, the ventral parts smooth. Petiole node: In profile, the top of the petiole node is straight, slightly pointed anteriorly. Petiole ventral lobe/Ventral projection (lamella): In profile, posteroventral part of petiole forms convex or sometimes partly straight (but never concave) line. Ventral postpetiolar process/Inferior tooth: Slightly rounded. Hairs: Erect hairs on petiole, postpetiole nodes and ventral postpetiolar process. Petiole width vs. postpetiole width (PEW/PPW): 0.688±0.025 [0.626, 0.722].

Gaster.

Color: Dark brown, first tergite usually pale brown at base. Hairs: Erect hairs all over the gaster. Sculpture: Smooth, shiny.

Geographic distribution

**Fig 7:**
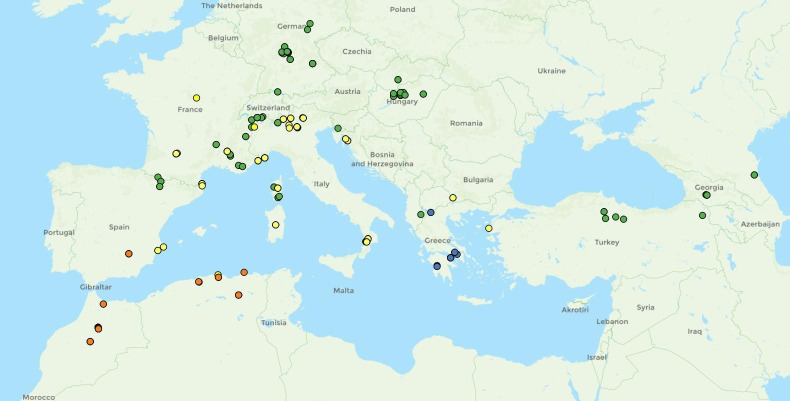
Distribution of *Temnothorax adlerzi* (blue dot), *T*. *algerianus* (orange dot), *T*. *kraussei* (yellow dot) and *T*. *ravouxi* (green dot).

This species is known only from some parts of northern and southern Greece (Fig 7) [33, 46].

Host ant usage

All samples investigated used the host species *Temnothorax exilis* (Emery, 1869) (n = 12) and no other host species is mentioned in the literature [31, 33].

Differential diagnosis

**Fig 8:**
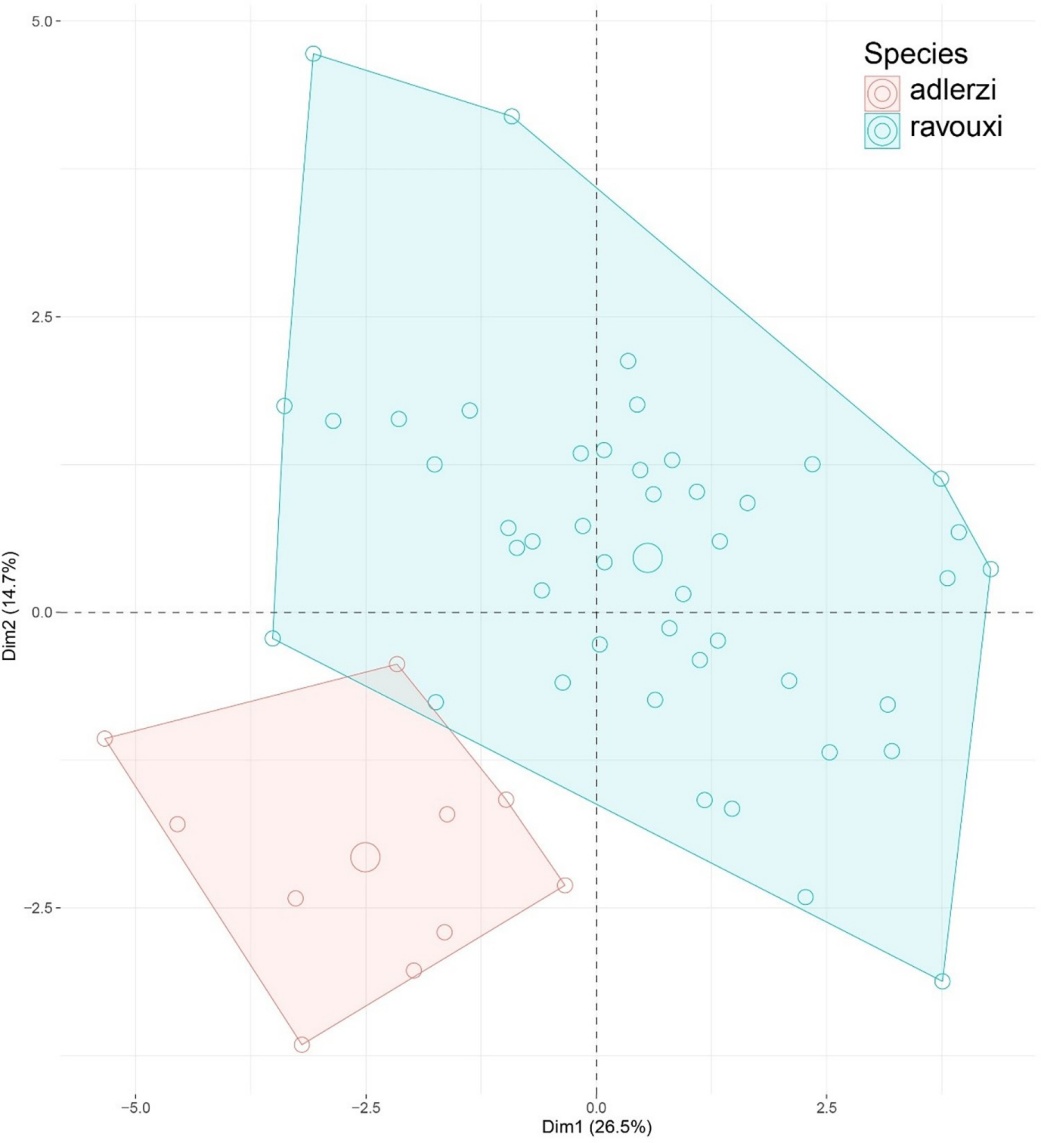
PCA plot of morphometric data of *Temnothorax adlerzi* (red), *T*. *ravouxi* (blue) nest samples illustrated on two principal components (Dim 1, Dim 2).

This is a workerless social parasite, only gynes have been found in nature, though in laboratory conditions a single worker is reported to have developed [33]. Salient external morphological features help separating the gynes of *T*. *adlerzi* from every species in this revisionary but *T*. *ravouxi*. These two species are very similar to each other. Morphometry yields an almost flawless separation for nest sample means (Fig 8), and a combination of five morphometric traits (D5a = +0.047*CW -0.073*FR -0.035*SPWI -0.018*ML +0.057*SPST +2.974) is necessary to separate individual gynes. This function yields a 94.5% classification success rate. The range of discriminant D5a scores for gynes are as follows:

*T*. *adlerzi* gynes D5a (n = 17) = +1.699 [-0.160, +3.894]

*T*. *ravouxi* gynes D5a (n = 110) = -1.699 [-4.064, +1.113]

### *Temnothorax algerianus* (Cagniant, 1968)

*Epimyrma algeriana* Cagniant [47]: 157 (w.q.m.) ALGERIA.

Combination in *Myrmoxenus*: Cagniant [48]: 198

Combination in *Temnothorax*: Ward et al. [29]: 75.

Type material investigated.

Two syntype *Epimyrma algeriana* workers were investigated from the type locality: Algeria: Atlas de Bleda, road to Station de Chréa, leg. presumably H. Cagniant (2w-HCPC).

**Fig 9:**
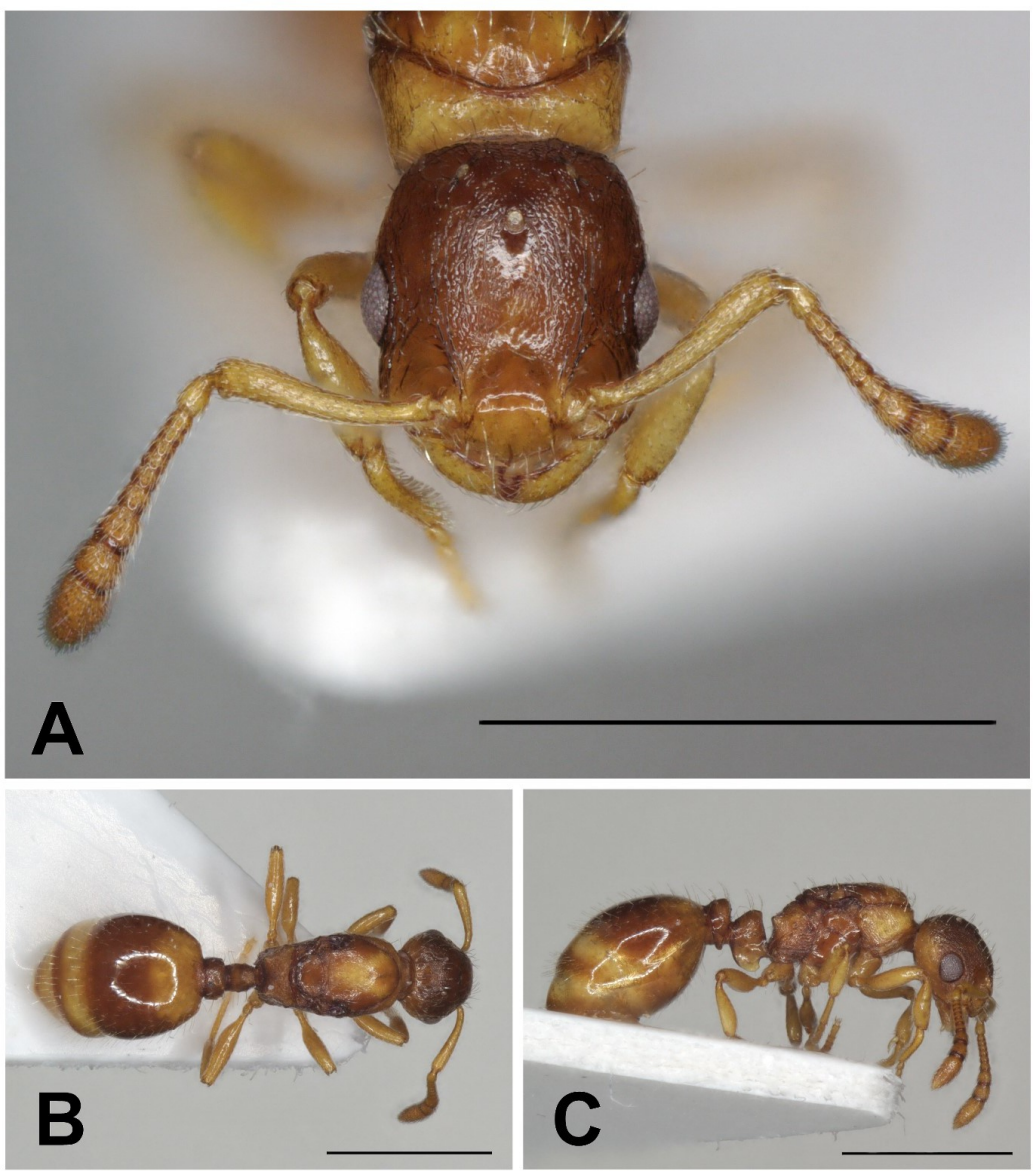
*Temnothorax algerianus* gyne. Head in full-face view (A), dorsal view of the body (B), lateral view of the body (C), scale bar: 1 mm.

Description of gynes. (Fig 9 and Table 4).

Body color brown. Body color pattern concolorous.

Head.

Absolute cephalic size: 615±18.398 [552, 649]. Cephalic length vs. maximum width of head capsule (CL/CWb): 1.184±0.019 [1.151, 1.225]. Color: Dark brown. Frons: Dark brown. Vertex: Dark brown. Scape: Pale brown. Antennal club: Brown. Head frontal sculpture: rugoso-reticulate with areolate ground sculpture. Moderately shiny. Clypeus: Smooth, shiny, with 1–2 faint longitudinal ridges. Mandibles: Smooth, 4–5 teeth, at long, t2 moderately long, t3-4 short, bt short. Anterior margin of clypeus: Evenly convex, slightly pointed. Hairs: Hairs erect allover except adpressed on the antennae. Postocular distance vs. cephalic length (POC/CL): 0.373±0.007 [0.356, 0.390]. Eye vs. absolute cephalic size (EYE/CS): 0.274±0.007 [0.257, 0.291]. Frontal carina distance vs. absolute cephalic size (FR/CS): 0.357±0.013 [0.328, 0.380]. Scape length vs. absolute cephalic size (SL/CL): 0.681±0.014 [0.649, 0.702].

Alitrunk.

Color and color pattern: Pale brown usually with irregular darker margins laterally and characteristic dark margins along anterior margin of mesonotum and longitudinal spot on each side. Mesoscutellum at least partly dark brown. Sculpture: Dorsal region of mesosoma rugulose with rugoso-areolate ground sculpture. Lateral region of pronotum rugoso-areolate. Anepisternum areolate lower third smooth, shiny. Katepisternum smooth, shiny, upper third areolate.r Mesopleuron areolate. Propodeal spines: Short, slightly rounded, triangulate. Hairs: Sparse erect hairs on pronotum, mesonotum and scutellum. Mesosoma length vs. absolute cephalic size (ML/CS): 1.458±0.034 [1.388, 1.503]. Maximum mesosoma width vs. absolute cephalic size (MW/CS): 0.842±0.029 [0.795, 0.942]. Spine length vs. absolute cephalic size (SPST/CS): 0.250±0.013 [0.209, 0.275]. Minimum spine distance at its base vs. absolute cephalic size (SPBA/CS): NA. Maximum spine distance at its tip vs. absolute cephalic size (SPWI/CS): 0.407±0.015 [0.374, 0.443]. Apical spine distance vs. absolute cephalic size (SPTI/CS): 0.368±0.010 [0.340, 0.383].

Pedicel.

Color: Brown, pale brown. Sculpture: Petiole and postpetiole nodes areolate, the ventral parts smooth. Petiole node: In profile, the top of petiole node is slightly or strongly convex and curves posteriorly. Petiole ventral lobe/Ventral projection (lamella): In profile, posteroventral part of petiole forms strongly convex (but never concave) line. Ventral postpetiolar process/Inferior tooth: Slightly rounded. Hairs: Erect hairs on petiole, postpetiole nodes and ventral pp process. Petiole width vs. postpetiole width (PEW/PPW): 0.701±0.031 [0.639, 0.791].

Gaster.

Color: Brown, first tergite usually pale brown at base. Hairs: Erect hairs all over the gaster. Sculpture: Smooth, shiny.

**Fig 10:**
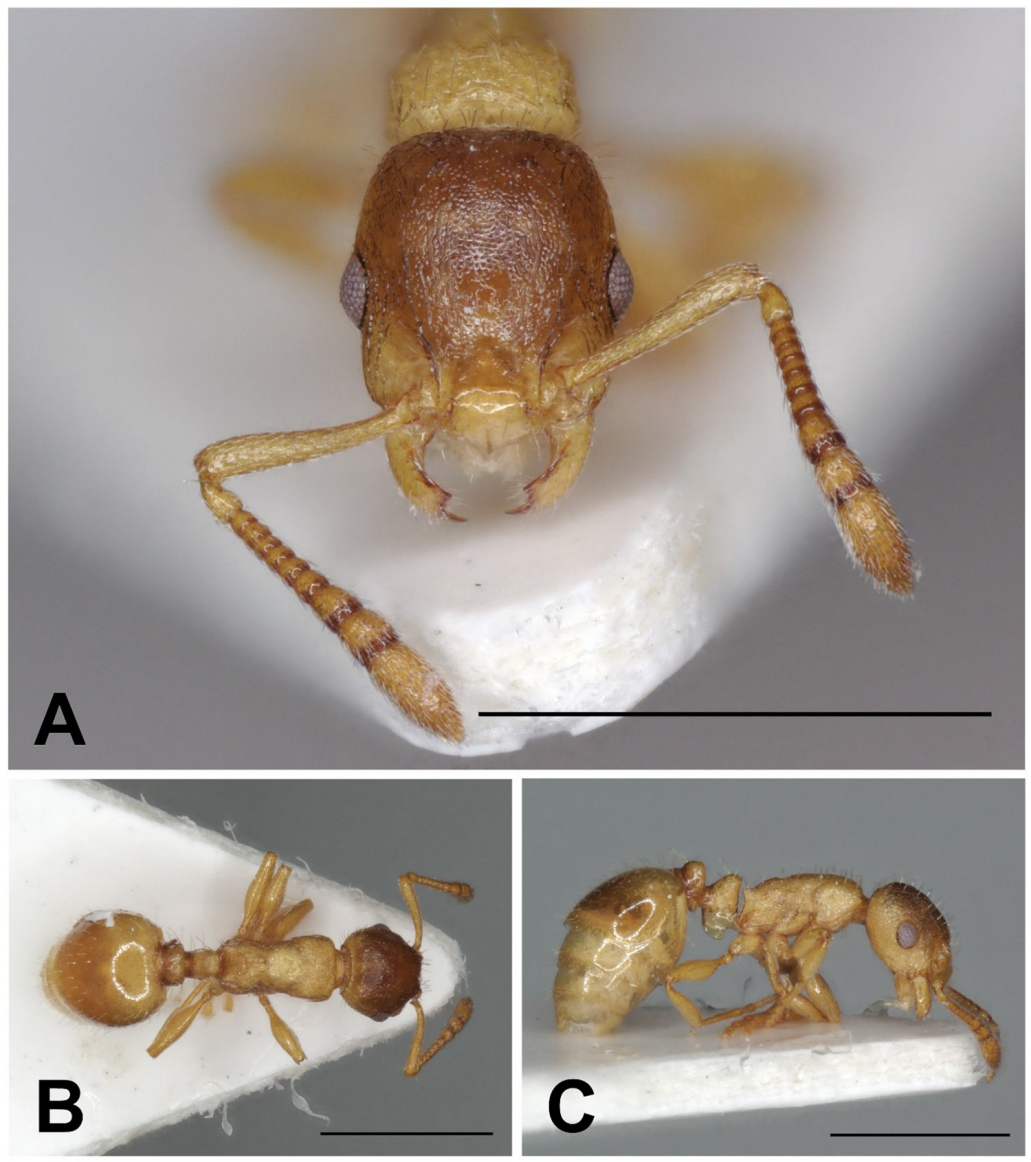
*Temnothorax algerianus* worker. Head in full-face view (A), dorsal view of the body (B), lateral view of the body (C), scale bar: 1 mm.

Description of workers. (Fig 10 and Table 5).

**Table 5:** Mean of morphometric ratios calculated based on worker individuals.

	*algerianus* (n = 44)	*bernardi* (n = 10)	*gordiagini* (n = 1)	*kraussei* (n = 70)	*microcellatus* (n = 51)	*ravouxi* (n = 174)	*stumperi* (n = 35)
**CS**	598±21.265	564±15.214	579	599±19.488	590±20.033	614±26.021	582±16.108
** **	[548, 639]	[545, 587]		[561, 650]	[546, 654]	[551, 665]	[551, 618]
**CL/CWb**	1.232±0.019	1.243±0.023	1.162	1.268±0.022	1.192±0.020	1.232±0.027	1.143±0.020
** **	[1.180, 1.263]	[1.199, 1.270		[1.220, 1.319]	[1.144, 1.230]	[1.155, 1.323]	[1.110, 1.188]
**POC/CL**	0.372±0.010	0.368±0.017	0.372	0.354±0.009	0.369±0.019	0.368±0.010	0.358±0.013
** **	[0.352, 0.398]	[0.344, 0.406]		[0.330, 0.371]	[0.348, 0.493]	[0.342, 0.407]	[0.335, 0.395]
**SL/CL**	0.702±0.015	0.714±0.031	0.723	0.706±0.018	0.734±0.018	0.672±0.022	0.703±0.030
** **	[0.668, 0.727]	[0.677, 0.781]		[0.670, 0.748]	[0.699, 0.783]	[0.613, 0.733]	[0.561, 0.747]
**FR/CS**	0.356±0.013	0.362±0.009	0.420	0.337±0.009	0.361±0.008	0.362±0.014	0.336±0.012
** **	[0.325, 0.383]	[0.344, 0.378]		[0.316, 0.362]	[0.337, 0.374]	[0.328, 0.406]	[0.313, 0.364]
**MW/CS**	0.661±0.021	0.684±0.021	0.695	0.623±0.016	0.663±0.012	0.672±0.022	0.706±0.017
** **	[0.629, 0.734]	[0.666, 0.727]		[0.592, 0.671]	[0.642, 0.705]	[0.619, 0.731]	[0.663, 0.735]
**SPBA/CS**	0.355±0.013	0.348±0.018	0.302	0.348±0.017	0.312±0.016	0.356±0.021	0.314±0.015
** **	[0.335, 0.405]	[0.313, 0.377]		[0.311, 0.407]	[0.285, 0.369]	[0.296, 0.405]	[0.279, 0.345]
**SPWI/CS**	0.367±0.016	0.387±0.015	0.290	0.381±0.025	0.328±0.015	0.378±0.026	0.332±0.021
** **	[0.342, 0.416]	[0.363, 0.411]		[0.326, 0.440]	[0.296, 0.373]	[0.309, 0.459]	[0.291, 0.389]
**SPTI/CS**	0.349±0.017	0.360±0.017	0.258	0.365±0.024	0.308±0.016	0.357±0.027	0.303±0.020
** **	[0.322, 0.393]	[0.329, 0.388]		[0.313, 0.427]	[0.277, 0.354]	[0.278, 0.435]	[0.261, 0.355]
**PEW/CS**	0.295±0.015	0.328±0.025	0.291	0.285±0.018	0.287±0.012	0.313±0.017	0.279±0.009
** **	[0.274, 0.349]	[0.266, 0.356]		[0.258, 0.355]	[0.264, 0.314]	[0.268, 0.356]	[0.254, 0.292]
**PPW/CS**	0.423±0.020	0.497±0.016	0.442	0.437±0.020	0.438±0.016	0.456±0.021	0.438±0.014
** **	[0.388, 0.496]	[0.468, 0.517]		[0.398, 0.500]	[0.405, 0.474]	[0.410, 0.517]	[0.405, 0.470]
**ML/CS**	1.232±0.030	1.252±0.033	1.292	1.210±0.034	1.251±0.026	1.274±0.037	1.273±0.022
** **	[1.171, 1.338]	[1.212, 1.330]		[1.154, 1.323]	[1.203, 1.300]	[1.160, 1.370]	[1.224, 1.315]
**SPST/CS**	0.225±0.013	0.239±0.013	0.280	0.256±0.018	0.227±0.014	0.262±0.021	0.244±0.014
** **	[0.200, 0.253]	[0.220, 0.260]		[0.210, 0.301]	[0.190, 0.266]	[0.215, 0.316]	[0.219, 0.268]
**PLST/CS**	0.362±0.013	0.367±0.021	0.411	0.355±0.019	0.392±0.015	0.376±0.019	0.372±0.015
** **	[0.332, 0.385]	[0.346, 0.423]		[0.321, 0.416]	[0.363, 0.422]	[0.310, 0.441]	[0.340, 0.411]
**PEH/CS**	0.574±0.019	0.609±0.028	0.560	0.571±0.025	0.570±0.017	0.621±0.031	0.594±0.015
** **	[0.531, 0.639]	[0.563, 0.668]		[0.524, 0.651]	[0.534, 0.603]	[0.547, 0.751]	[0.572, 0.623]
**SPH/CS**	0.268±0.013	0.295±0.019	0.280	0.285±0.016	0.317±0.029	0.306±0.021	0.291±0.017
** **	[0.247, 0.311]	[0.266, 0.331]		[0.257, 0.337]	[0.278, 0.379]	[0.254, 0.359]	[0.260, 0.331]
**PPH/CS**	0.468±0.014	0.500±0.027	0.458	0.465±0.018	0.419±0.014	0.487±0.021	0.465±0.015
** **	[0.439, 0.499]	[0.444, 0.533]		[0.427, 0.531]	[0.395, 0.458]	[0.426, 0.543]	[0.435, 0.514]
**EYE/CS**	0.244±0.008	0.258±0.021	0.265	0.236±0.008	0.258±0.010	0.250±0.009	0.253±0.010
	[0.230, 0.262]	[0.200, 0.272]		[0.217, 0.250]	[0.237, 0.283]	[0.226, 0.280]	[0.229, 0.275]

Morphometric traits are divided by cephalic size (CWb), ±SD are provided in the upper row, minimum and maximum values are given in parentheses in the lower row.

Body color brown, pale brown. Body color pattern concolorous.

Head.

Absolute cephalic size: 598±21.265 [548, 639]. Cephalic length vs. maximum width of head capsule (CL/CWb): 1.232±0.019 [1.180, 1.263]. Color: Brown, pale brown. Frons: Brown. Vertex: Brown. Scape: Pale brown. Antennal club: Brown. Head frontal sculpture: Areolate, moderately shiny. Clypeus: Smooth, shiny with very few longitudinal ridge. Mandibles: Smooth, shiny, 5 teeth, at long, t2 moderate, t3-4 short, bt medium. Anterior margin of clypeus: Evenly convex, slightly pointed. Hairs: Hairs erect allover except adpressed on the antennae. Postocular distance vs. cephalic length (POC/CL): 0.372±0.010 [0.352, 0.398]. Eye vs. absolute cephalic size (EYE/CS): 0.244±0.008 [0.230, 0.262]. Frontal carina distance vs. absolute cephalic size (FR/CS): 0.356±0.013 [0.325, 0.383]. Scape length vs. absolute cephalic size (SL/CL): 0.702±0.015 [0.668, 0.727].

Alitrunk.

Color and color pattern: Pale brown. Sculpture: Dorsal region of mesosoma rugulose with areolate ground sculpture. Lateral region of pronotum areolate ground sculpture, main sculpture forked costate. Mesopleuron areolate ground sculpture superimposed by dispersed rugulae. Metapleuron areolate ground sculpture superimposed by dispersed rugulae. Propodeal spines: Short, pointed, triangulate. Hairs: Sparse erect hairs mainly on dorsal face of pronotum and mesonotum. Mesosoma length vs. absolute cephalic size (ML/CS): 1.232±0.030 [1.171, 1.338]. Maximum mesosoma width vs. absolute cephalic size (MW/CS): 0.661±0.021 [0.629, 0.734]. Spine length vs. absolute cephalic size (SPST/CS): 0.225±0.013 [0.200, 0.253]. Minimum spine distance at its base vs. absolute cephalic size (SPBA/CS): 0.355±0.013 [0.335, 0.405]. Maximum spine distance at its tip vs. absolute cephalic size (SPWI/CS): 0.367±0.016 [0.342, 0.416]. Apical spine distance vs. absolute cephalic size (SPTI/CS): 0.349±0.017 [0.322, 0.393].

Pedicel.

Color: Pale brown. Sculpture: Petiole and postpetiole nodes areolate, the ventral parts smooth. Petiole node: In side view, Dorsal face of petiole node curving backwards without angle. Petiole ventral lobe/Ventral projection (lamella): In side view, posteroventral part of petiole forms convex or sometimes partly straight (but never concave) line. Ventral postpetiolar process/Inferior tooth: Slightly rounded. Hairs: Erect hairs on petiole and postpetiole nodes. Petiole width vs. Postpetiole width (PEW/PPW): 0.697±0.037 [0.611, 0.854].

Gaster.

Color: Brown, pale brown, first tergite brown, usually pale brown at base. t2-3-4-5 pale brown. Hairs: Erect hairs all over the gaster. Sculpture: Smooth, shiny.

Differential diagnosis

**Fig 11:**
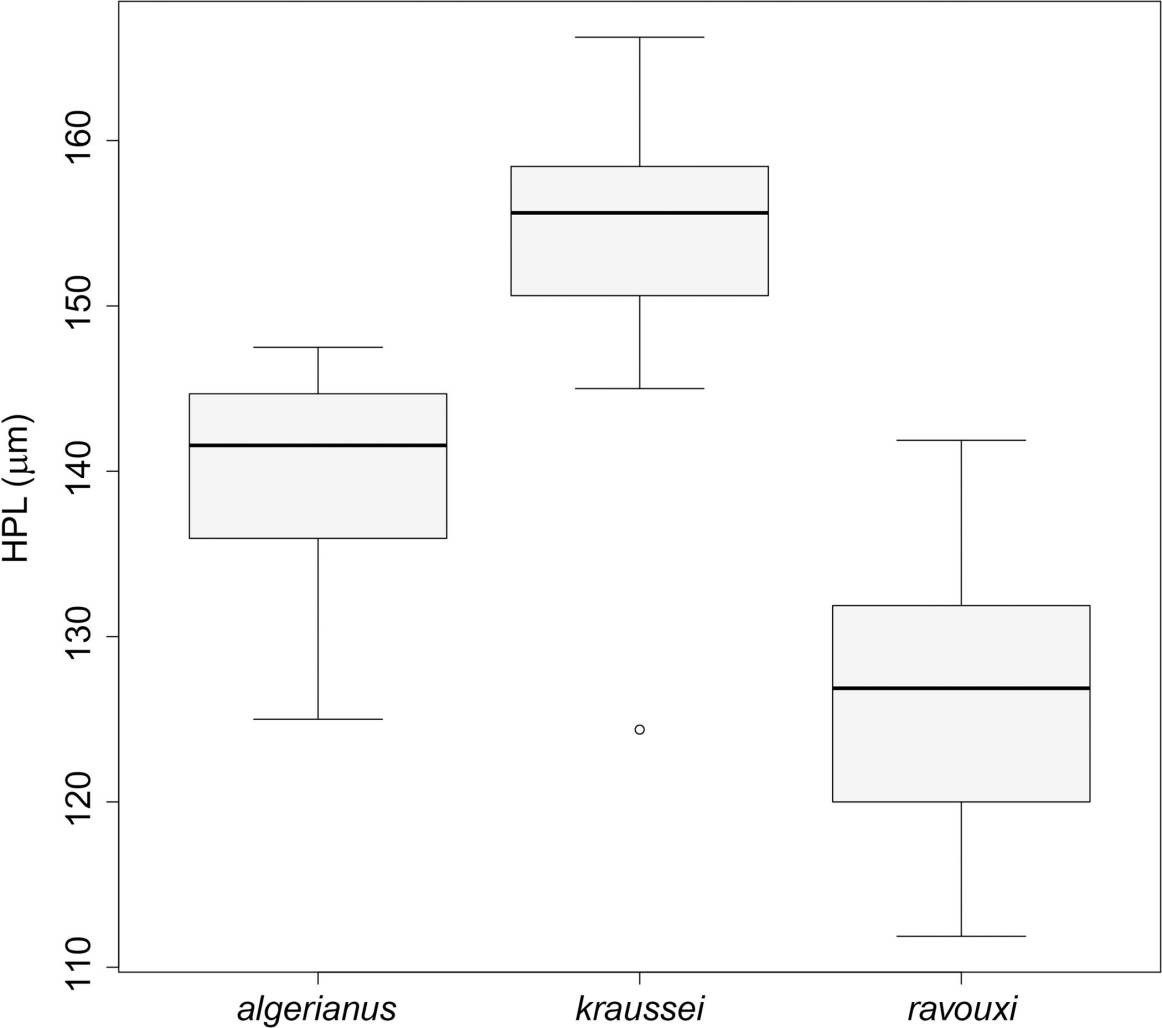
The lengths of the longest hairs on the petiole of *T*. *algerianus*, *T*. *kraussei* and *T*. *ravouxi* gynes.

**Fig 12:**
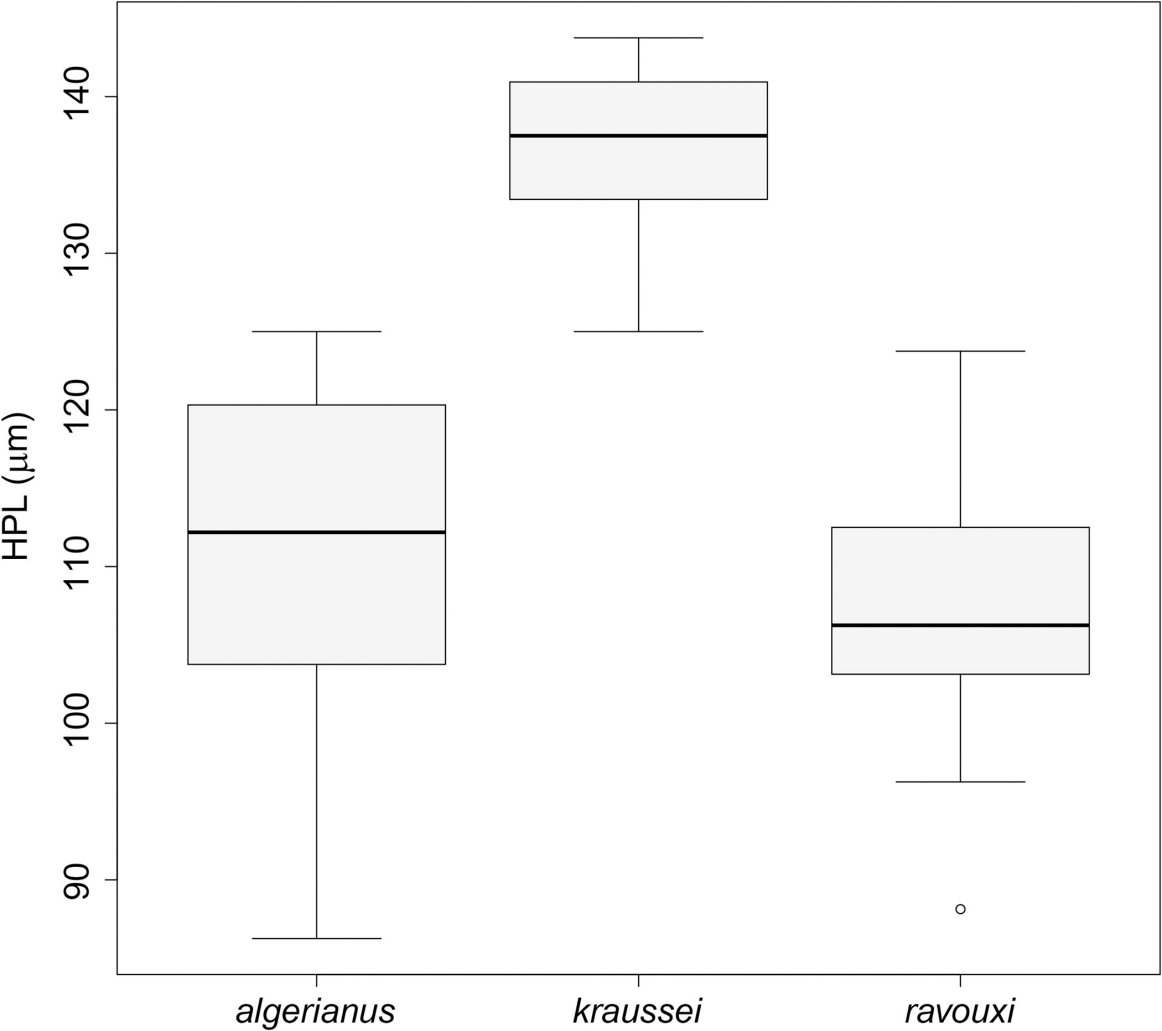
The lengths of the longest hairs on the petiole of *T*. *algerianus*, *T*. *kraussei* and *T*. *ravouxi* workers.

Both workers and gynes of this species are very similar to *T*. *kraussei* and *T*. *ravouxi* in shape, sculpture and morphometric characteristics. Dorsal setae on the petiole help to distinguish *T*. *algerianus* (and *T*. *ravouxi*) from *T*. *kraussei* in both castes; the latter has much longer hairs. In gynes, the hair length narrowly overlapping between *T*. *kraussei* and *T*. *algerianus* (Fig 11); the former has longer hairs (140μm to 175μm), *T*. *algerianus* gynes bear shorter pilosity (125μm to 145μm). The longest hair on the pedicel of *T*. *kraussei* workers exceeds 130μm [125μm, 147μm], while the range of the longest hair length in *T*. *algerianus* is between 85 to 130μm (Fig 12). Note, the hairs in *T*. *ravouxi* workers are similarly short, ranging from 88 to 124μm.

Separating individual gynes and workers of *T*. *algerianus* from *T*. *ravouxi* in case of questionable cases require a combination of multiple traits. For gynes a combination of three morphometric traits (D3 = +0.117*ELmax -0.046*SL +0.038*SPH -9.973) is necessary. This function yields a 97.3% classification success rate in gynes. The range of discriminant D3 scores for individual gynes are as follows:

*T*. *algerianus* gynes D3 (n = 34) = -1.882 [-3.857, +1.046]

*T*. *ravouxi* gynes D3 (n = 113) = +1.882 [-1.082, +5.154]

Separating individual workers requires a combination of five traits (D5b = +0.029*SL +0.080*SPBA -0.070*PPW -0.045*SPH -0.056*ELmax +5.703). This combination provides a 96.3% classification success rate in individual workers. The range of discriminant D5b scores for workers are as follows:

*T*. *algerianus* workers D5b (n = 45) = +1.673 [-0.747, +3.250]

*T*. *ravouxi* workers D5b (n = 173) = -1.673 [-4.500, +0.595]

Geographic distribution

The species was previously known only from North Africa in Morocco and Algeria, where it is quite common. Based on two samples we examined, the species also occurs in southern Spain near the village of Hornos (Fig 7) [23, 46, 47].

Host ant usage

With the exception of two samples from Spain (Spain: Hornos), where *Temnothorax racovitzai* (Bondroit, 1918) and *T*. *unifasciatus* was the host, all samples investigated used *Temnothorax spinosus* (Forel, 1909) as host species (n = 15). The following host species are also mentioned in the literature: *Temnothorax curtulus* (Santschi, 1929), *T*. *gentilis* (Santschi, 1923), *T*. *monjauzei* (Cagniant, 1968) and *T*. *tebessae* (Forel, 1890) [23, 31, 47].

### *Temnothorax bernardi* (Espadaler, 1982)

*Epimyrma bernardi* Espadaler [49]: 1 (w.q.) SPAIN.

Combination in *Temnothorax*: Ward et al. [29]: 75.

Type material investigated.

One paratype worker was investigated from type locality: Spain: Avila, Sierra de Gredos, 1400 m., 22-23.vii.1979 (1w, HCPC).

**Fig 13:**
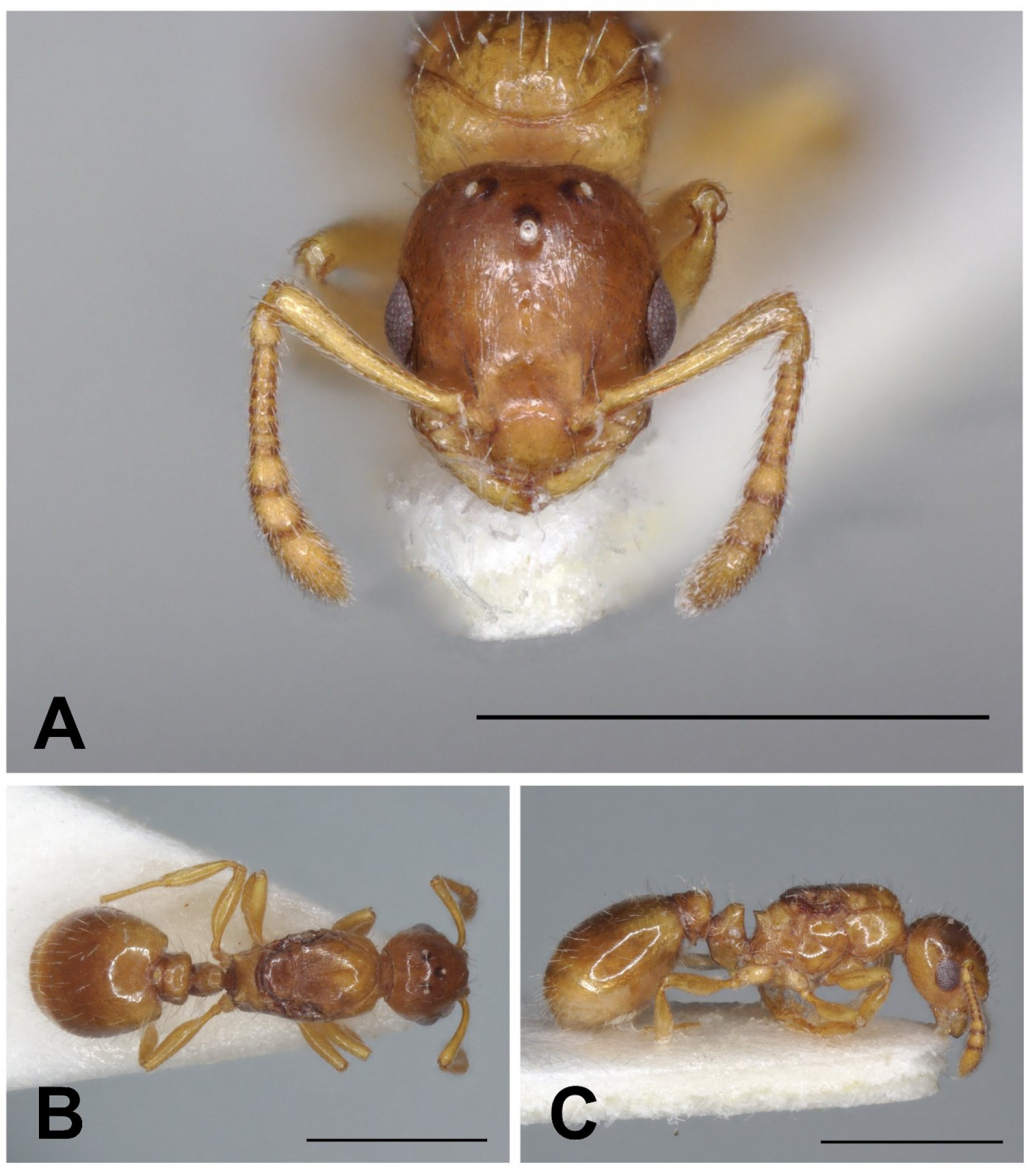
*Temnothorax bernardi* gyne. Head in full-face view (A), dorsal view of the body (B), lateral view of the body (C), scale bar: 1 mm.

Description of gynes (Fig 13 and Table 4).

Body color brown, pale brown. Body color pattern concolorous.

Head.

Absolute cephalic size: 589±10.618 [576, 609]. Cephalic length vs. maximum width of head capsule (CL/CWb): 1.186±0.013 [1.163, 1.205]. Color: Brown. Frons: Brown. Vertex: Brown. Scape: Pale brown. Antennal club: Pale brown. Head frontal sculpture: Extensively smooth, shiny with very feeble or missing sculpture. Clypeus: Smooth and shiny. Mandibles: Smooth. Anterior margin of clypeus: Evenly convex. Hairs: Hairs erect allover except adpressed on the antennae. Postocular distance vs. cephalic length (POC/CL): 0.348±0.005 [0.341, 0.355]. Eye vs. absolute cephalic size (EYE/CS): 0.299±0.005 [0.290, 0.306]. Frontal carina distance vs. absolute cephalic size (FR/CS): 0.366±0.007 [0.351, 0.372]. Scape length vs. absolute cephalic size (SL/CL): 0.694±0.012 [0.680, 0.708].

Alitrunk.

Color and color pattern: Pale brown, Mesoscutellum brown. Sculpture: Dorsal region of mesosoma extensively smooth with very feeble parallel costulate or missing sculpture. Lateral region of pronotum smooth, shiny. Anepisternum smooth, shiny. Katepisternum smooth, shiny, upper third areolate. Propodeal spines: Short, slightly rounded, triangulate. Hairs: Relatively dense erect hairs especially on pro- and mesonotum and on the posterior face of propodeum. Mesosoma length vs. absolute cephalic size (ML/CS): 1.489±0.014 [1.474, 1.517]. Maximum mesosoma width vs. absolute cephalic size (MW/CS): 0.878±0.033 [0.850, 0.944]. Spine length vs. absolute cephalic size (SPST/CS): 0.247±0.009 [0.238, 0.258]. Minimum spine distance at its base vs. absolute cephalic size (SPBA/CS): NA. Maximum spine distance at its tip vs. absolute cephalic size (SPWI/CS): 0.455±0.010 [0.437, 0.467]. Apical spine distance vs. absolute cephalic size (SPTI/CS): 0.413±0.007 [0.400, 0.422].

Pedicel.

Color: Brown, pale brown. Sculpture: Petiole and postpetiole nodes smooth and shiny. Petiole node: In profile, dorsal face of petiole node curving backwards without angle. Petiole ventral lobe/Ventral projection (lamella): In profile, posteroventral part of petiole forms convex or sometimes partly straight (but never concave) line. Ventral postpetiolar process/Inferior tooth: Slightly pointed. Hairs: Erect hairs on petiole, postpetiole nodes and ventral pp process. Petiole width vs. Postpetiole width (PEW/PPW): 0.642±0.019 [0.620, 0.668].

Gaster.

Color: Brown, first tergite usually pale brown at base. Hairs: Erect hairs all over the gaster. Sculpture: Smooth, shiny.

**Fig 14:**
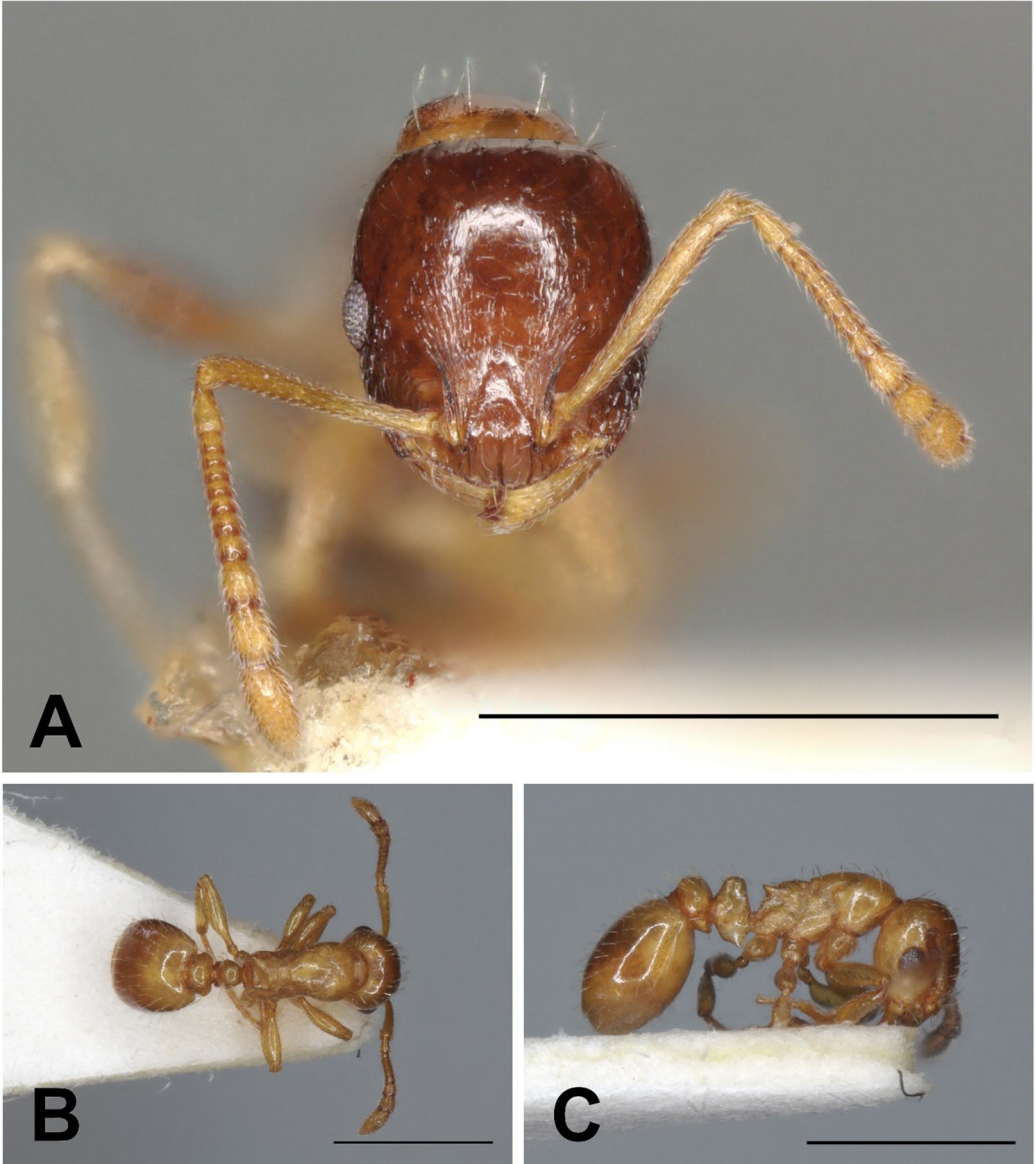
*Temnothorax bernardi* worker. Head in full-face view (A), dorsal view of the body (B), lateral view of the body (C), scale bar: 1 mm.

Description of workers (Fig 14 and Table 5).

Body color brown, pale brown.

Head.

Absolute cephalic size: 564±15.214 [545, 587]. Cephalic length vs. maximum width of head capsule (CL/CWb): 1.243±0.023 [1.199, 1.270]. Color: Brown, pale brown. Frons: Brown. Vertex: Brown. Scape: Pale brown. Antennal club: Pale brown. Head frontal sculpture: Extensively smooth, shiny with very feeble or missing sculpture. Clypeus: Smooth, shiny, with very few fainth longitudinal ridges. Mandibles: Smooth, shiny, 4 teeth at long, t2 moderate, t-3 bt very short. Anterior margin of clypeus: Evenly convex, slightly pointed. Hairs: Hairs erect allover except adpressed on the antennae. Postocular distance vs. cephalic length (POC/CL): 0.368±0.017 [0.344, 0.406]. Eye vs. absolute cephalic size (EYE/CS): 0.258±0.021 [0.200, 0.272]. Frontal carina distance vs. absolute cephalic size (FR/CS): 0.362±0.009 [0.344, 0.378]. Scape length vs. absolute cephalic size (SL/CL): 0.714±0.031 [0.677, 0.781].

Alitrunk.

Color and color pattern: Pale brown. Sculpture: Dorsal region of mesosoma extensively smooth with very feeble or missing sculpture. Lateral region of pronotum sculpture: Extensively smooth, very feeble areolate or missing sculpture. Mesopleuron extensively smooth, very feeble aerolate or missing sculpture. Metapleuron extensively smooth, very feeble aerolate or missing sculpture. Propodeal spines: Short, pointed, triangulate. Hairs: Sparse erect hairs mainly on dorsal face of pronotum, mesonotum and propodeum. Mesosoma length vs. absolute cephalic size (ML/CS): 1.252±0.033 [1.212, 1.330]. Maximum mesosoma width vs. absolute cephalic size (MW/CS): 0.684±0.021 [0.666, 0.727]. Spine length vs. absolute cephalic size (SPST/CS): 0.239±0.013 [0.220, 0.260]. Minimum spine distance at its base vs. absolute cephalic size (SPBA/CS): 0.348±0.018 [0.313, 0.377]. Maximum spine distance at its tip vs. absolute cephalic size (SPWI/CS): 0.387±0.015 [0.363, 0.411]. Apical spine distance vs. absolute cephalic size (SPTI/CS): 0.360±0.017 [0.329, 0.388].

Pedicel.

Color: Pale brown. Sculpture: Petiole and postpetiole nodes feeble areolate or missing sculpture, ventral parts smooth. Petiole node: In side view, dorsal face of petiole node curving backwards without angle. Petiole ventral lobe/Ventral projection (lamella): In side view, posteroventral part of petiole forms convex or sometimes partly straight or slightly concave line. Ventral postpetiolar process/Inferior tooth: Highly pointed. Hairs: Erect hairs on petiole, postpetiole nodes and ventral pp process. Petiole width vs. postpetiole width (PEW/PPW): 0.663±0.047 [0.548, 0.721].

Gaster.

Brown, pale brown, first tergite brown, usually pale brown at base. T2-3-4-5 pale brown. Hairs: Erect hairs all over the gaster. Sculpture: Smooth, shiny.

Diagnosis in key.

Geographic distribution

**Fig 15:**
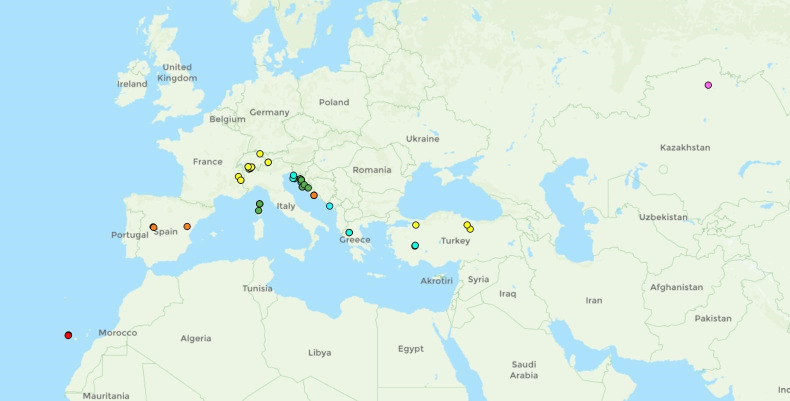
Distribution of *Temnothorax bernardi* (orange dot), *T*. *birgitae* (red dot), *T*. *corsicus* (green dot), *T*. *gordiagini* (pink dot), *T*. *microcellatus* (cyan dot), *T*. *stumperi* (yellow dot).

Only a few records of the species are known from the Iberian Peninsula (Spain) and the Western Balkans in Croatia (Fig 15) [46, 49, 50].

Host ant usage

With the exception of three samples from Croatia (Split), where *Temnothorax recedens* (Nylander, 1856) was the host, all samples investigated (n = 3) used *Temnothorax gredosi* (Espadaler & Collingwood, 1982) as host species and no other host species is mentioned in the literature: [31, 49].

### *Temnothorax birgitae* (Schulz, 1994)

*Epimyrma birgitae* Schulz [42]: 432 (q.m.) SPAIN (Canary Is).

Combination in *Temnothorax*: Ward et al. [29]: 15.

Type material investigated.

Five paratype gynes were investigated from the type locality: Spain: Canary Is., N side Tenerife, 9 km. SSE Puerto de la Cruz, Valle de la Orotava, ca 1200 m., 25.VI.1990, leg. A. Schulz (2g-ZMLS, 3g-SMNG).

**Fig 16:**
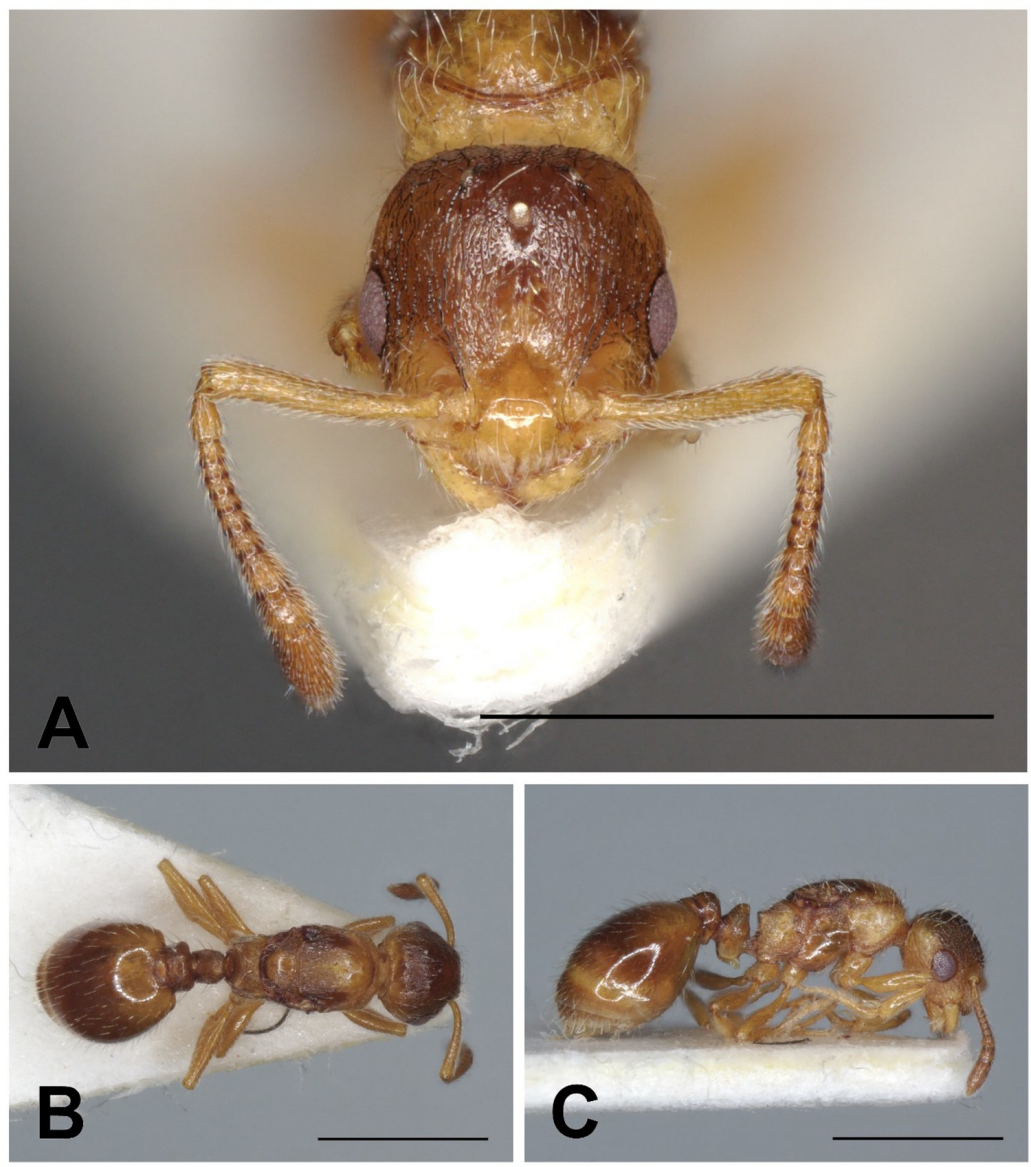
*Temnothorax birgitae* gyne. Head in full-face view (A), dorsal view of the body (B), lateral view of the body (C), scale bar: 1 mm.

Description of gynes (Fig 16 and Table 4).

Body color brown. Body color pattern concolorous.

Head

Absolute cephalic size: 589±24.456 [554, 634]. Cephalic length vs. maximum width of head capsule (CL/CWb): 1.161±0.020 [1.135, 1.192]. Color: Brown. Frons: Brown. Vertex: Dark brown. Scape: Pale brown, brown. Antennal club: Dark brown. Head frontal sculpture: Rugoso-reticulate with areolate ground sculpture. Shiny. Clypeus: Smooth with median and lateral ridges. Mandibles: Smooth, 4 teeth, at long, t2-3 and bt short. Anterior margin of clypeus: Evenly convex. Hairs: Hairs erect allover including scapes. Postocular distance vs. cephalic length (POC/CL): 0.376±0.014 [0.363, 0.400]. Eye vs. absolute cephalic size (EYE/CS): 0.274±0.014 [0.240, 0.284]. Frontal carina distance vs. absolute cephalic size (FR/CS): 0.341±0.016 [0.305, 0.356]. Scape length vs. absolute cephalic size (SL/CL): 0.718±0.013 [0.702, 0.738].

Alitrunk.

Color and color pattern: Pale brown usually with irregular darker margins laterally and characteristic dark margins along anterior margin of mesonotum and longitudinal spot on each side. Mesoscutellum at least partly dark brown. Sculpture: Dorsal region of mesosoma rugulose with rugoso-areolate ground sculpture. Lateral region of pronotum rugoso-areolate. Anepisternum areolate lower third smooth, shiny. Katepisternum smooth, shiny, upper third areolate. Metapleuron rugoso-areolate. Propodeal spines: Very short, dull, triangulate. Hairs: Relatively dense erect hairs especially on pro- and mesonotum and on the posterior face of propodeum. Mesosoma length vs. absolute cephalic size (ML/CS): 1.445±0.029 [1.410, 1.475]. Maximum mesosoma width vs. absolute cephalic size (MW/CS): 0.820±0.026 [0.791, 0.873]. Spine length vs. absolute cephalic size (SPST/CS): 0.263±0.009 [0.249, 0.275]. Minimum spine distance at its base vs. absolute cephalic size (SPBA/CS): NA. Maximum spine distance at its tip vs. absolute cephalic size (SPWI/CS): 0.406±0.020 [0.377, 0.437]. Apical spine distance vs. absolute cephalic size (SPTI/CS): 0.363±0.019 [0.337, 0.394].

Pedicel.

Color: Brown, pale brown. Sculpture: Petiole and postpetiole nodes rugoso-areolate, the ventral parts smooth. Petiole node: In side view, Dorsal face of petiole node curving backwards without angle. Petiole ventral lobe/Ventral projection (lamella): In side view, posteroventral part of petiole forms convex or sometimes partly straight line. In some specimens, the lamella has an elongated, rounded process. Ventral postpetiolar process/Inferior tooth: Slightly rounded. Hairs: Erect hairs on petiole, postpetiole nodes and ventral pp process. Petiole width vs. Postpetiole width (PEW/PPW): 0.675±0.015 [0.655, 0.701].

Gaster.

Color: Brown, first tergite usually pale brown at base. Hairs: Erect hairs all over the gaster. Sculpture: Smooth, shiny.

Diagnosis in key.

Geographic distribution

The species is endemic to the Canary Islands (Fig 15) [42, 46].

Host ant usage

Only the type series is known and investigated, the only host being *Temnothorax gracilicornis nivarianus* (Santschi, 1925) [42].

### *Temnothorax corsicus* (Emery, 1895)

*Formicoxenus corsicus* Emery [51]: 68 (q.) FRANCE (Corsica).

Combination in *Epimyrma*: Emery [18]: 262.

Combination in *Myrmoxenus*: Bračko [52]: 139.

Combination in *Temnothorax*: Ward et al. [29]: 75.

Type material investigated.

One holotype gyne was investigated from the type locality: France: Corsica, leg. de Saulcy (1g-MSNG, CASENT0904775).

**Fig 17:**
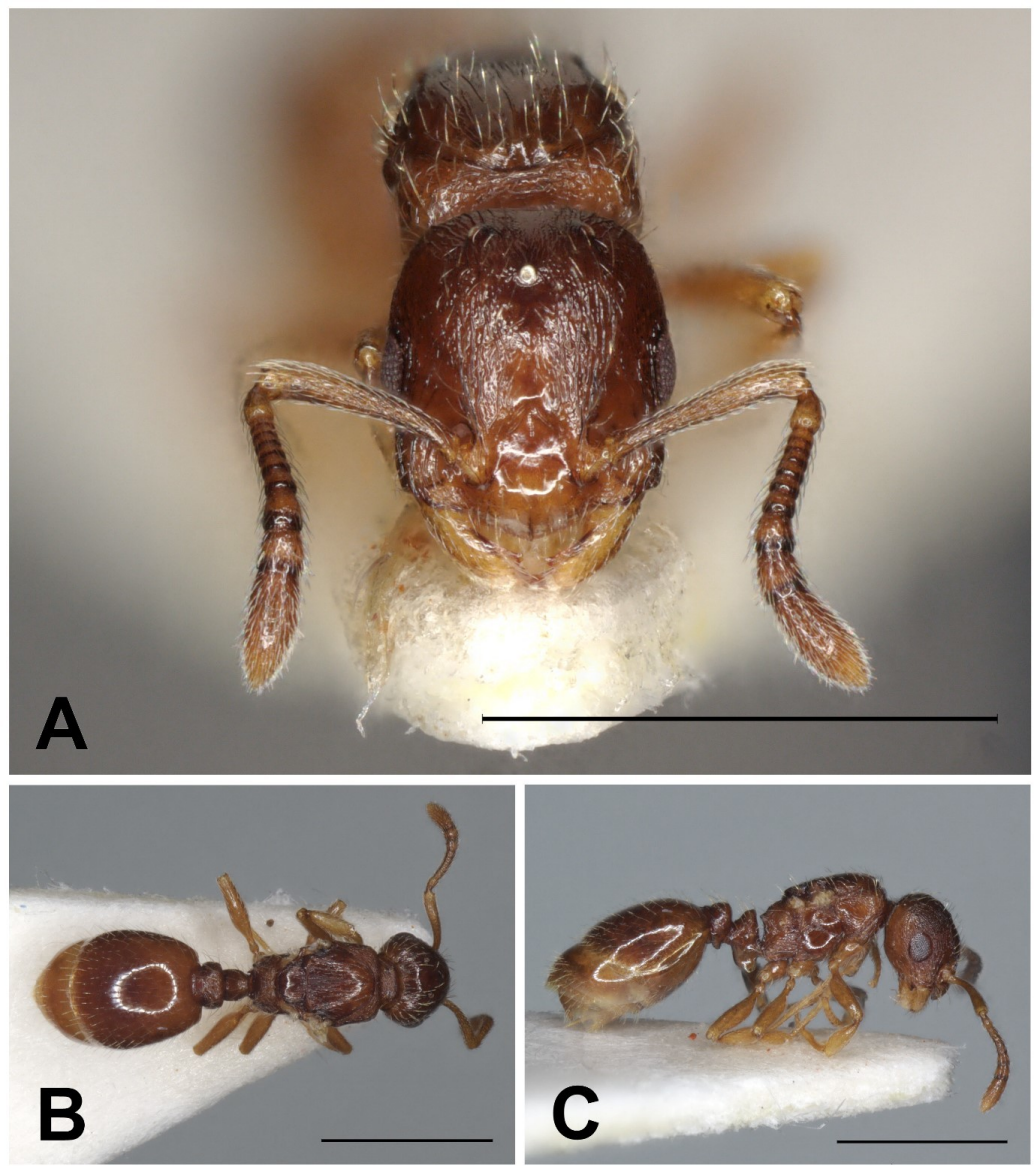
*Temnothorax corsicus* gyne. Head in full-face view (A), dorsal view of the body (B), lateral view of the body (C), scale bar: 1 mm.

Description of gynes (Fig 17 and Table 4).

Body color dark brown, dark amber-like. Body color pattern concolorous.

Head.

Absolute cephalic size: 593±18.745 [544, 640]. Cephalic length vs. maximum width of head capsule (CL/CWb): 1.207±0.024 [1.129, 1.266]. Color: Dark brown. Frons: Dark brown. Vertex: Dark brown. Scape: Brown. Antennal club: Brown, pale brown. Head frontal sculpture: Head frons medially shiny, with feeble costulate laterally. Clypeus: Smooth and shiny, with a few faint longitudinal ridges. Mandibles: Smooth, 4–5 teeth, at, t2 long, t3-bt small, rounded. Anterior margin of clypeus: Evenly convex, slightly pointed. Hairs: Hairs erect allover except adpressed on the antennae. Postocular distance vs. cephalic length (POC/CL): 0.356±0.012 [0.320, 0.384]. Eye vs. absolute cephalic size (EYE/CS): 0.265±0.009 [0.241, 0.285]. Frontal carina distance vs. absolute cephalic size (FR/CS): 0.364±0.009 [0.340, 0.388]. Scape length vs. absolute cephalic size (SL/CL): 0.625±0.017 [0.575, 0.658].

Alitrunk.

Color and color pattern: Brown, Dark brown, mesonotum and mesoscutellum often darker. Sculpture: Dorsal region of mesosoma extensively smooth with parallel costulate. Lateral region of pronotum extensively smooth, shiny with parallel costulate. Anepisternum feeble areolate, shiny. Katepisternum smooth, shiny, upper third areolate. Metapleuron rugoso-areolate. Propodeal spines: Very short, pointed, triangulate. Hairs: Relatively sparse erect hairs especially on pro- and mesonotum and on the posterior face of propodeum. Mesosoma length vs. absolute cephalic size (ML/CS): 1.379±0.040 [1.280, 1.465]. Maximum mesosoma width vs. absolute cephalic size (MW/CS): 0.796±0.038 [0.711, 0.886]. Spine length vs. absolute cephalic size (SPST/CS): 0.250±0.013 [0.207, 0.283]. Minimum spine distance at its base vs. absolute cephalic size (SPBA/CS): NA. Maximum spine distance at its tip vs. absolute cephalic size (SPWI/CS): 0.406±0.019 [0.345, 0.456]. Apical spine distance vs. absolute cephalic size (SPTI/CS): 0.366±0.024 [0.300, 0.414].

Pedicel.

Color: Brown, dark brown. Sculpture: Petiole and postpetiole nodes areolate, the ventral parts smooth. Petiole node: In profile, dorsal face of petiole node curving backwards with angle. Petiole ventral lobe/Ventral projection (lamella): In profile, posteroventral part of petiole forms convex or sometimes partly straight (but never concave) line. Ventral postpetiolar process/Inferior tooth: Slightly pointed. Hairs: Erect hairs on petiole, postpetiole nodes and ventral pp process. Petiole width vs. postpetiole width (PEW/PPW): 0.666±0.019 [0.625, 0.727].

Gaster.

Color: Brown, dark brown. Hairs: Erect hairs all over the gaster. Sculpture: Smooth, shiny.

Diagnosis in key.

Geographic distribution

The species is currently known from Corsica, Croatia (Fig 15) and France, Italy, and Serbia [46, 53, 54].

Host ant usage

Based on the analysis of 23 samples, the only known host was *Temnothorax exilis* (Emery, 1869), but no specimens of the host species were available for 7 samples. The only known host species mentioned in the literature is *T*. *exilis* [31, 53].

### *Temnothorax gordiagini* (Ruzsky, 1902)

*Myrmoxenus gordiagini* Ruzsky [55]: 475 (w.q.m.) KAZAKHSTAN.

Combination in *Epimyrma*: Bolton [43]: 188.

Combination in *Myrmoxenus*: Schulz & Sanetra [25]: 159.

Combination in *Temnothorax*: Ward et al. [29]: 75.

Type material investigated.

One lectotype *Myrmoxenus gordiagini* worker was investigated from the type locality: Kazakhstan: Akmola region, nr Kokshetau, 1896, leg. M. Ruzsky (1w-MSNG, CASENT0904773).

Description of gynes.

Syntype *M*. *gordiagini* gynes are supposed to be in the ZMUM, but according to information from the curator of the collection (Dr. Fedoseeva Elena Borisovna), these are not in this collection.

**Fig 18:**
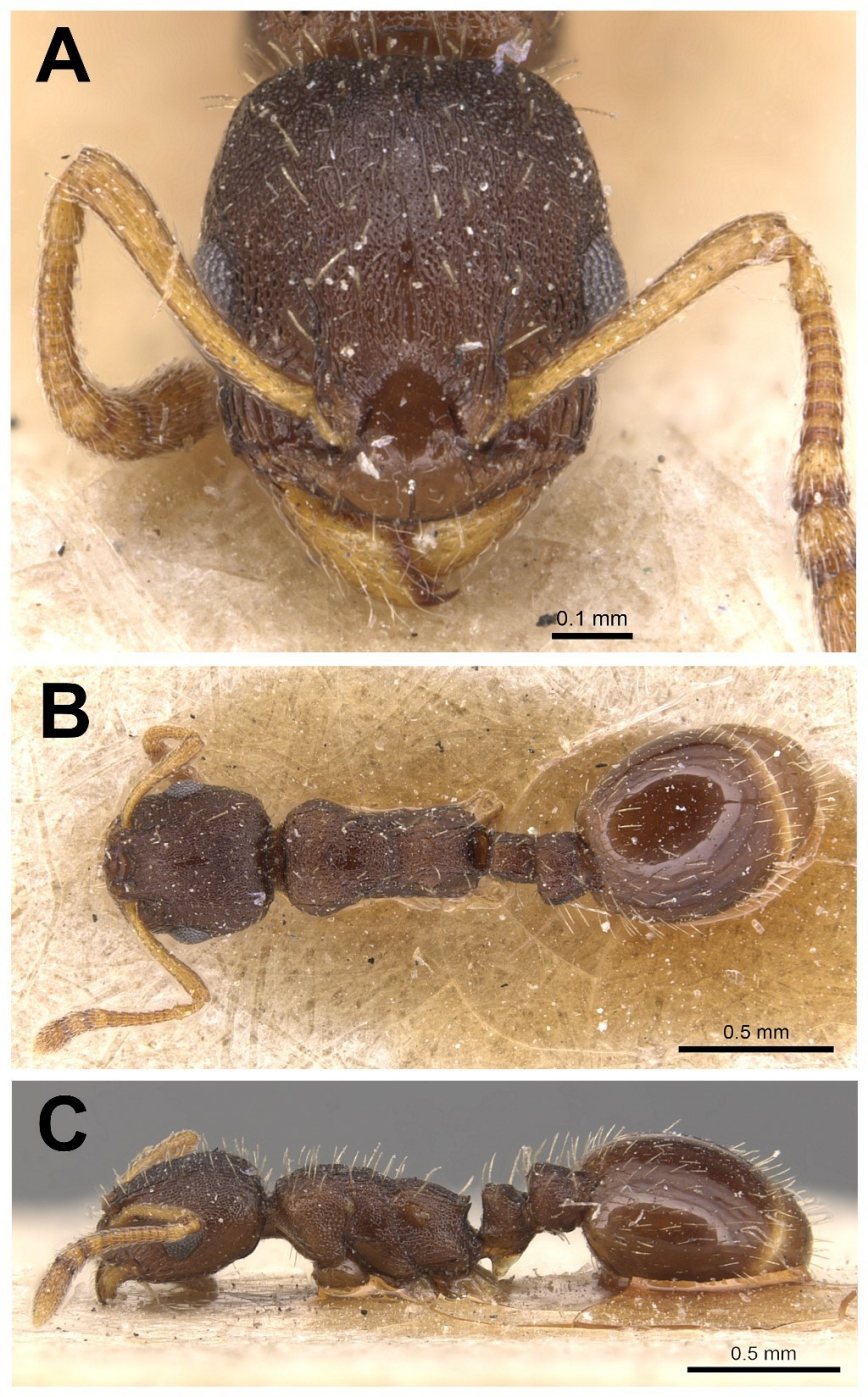
*Temnothorax gordiagini* worker. Head in full-face view (A), dorsal view of the body (B), lateral view of the body (C) The original images were taken by Will Ericson (Antweb.org).

Description of workers (Fig 18 and Table 5).

Body color dark brown. Body color pattern concolorous.

Head.

Absolute cephalic size: 579. Cephalic length vs. maximum width of head capsule (CL/CWb): 1.162. Color: Dark brown. Frons: Dark brown. Vertex: Dark brown. Scape: Pale brown. Antennal club: Pale brown. Head frontal sculpture: Reticulate with areolate ground sculpture. Moderately shiny. Clypeus: Smooth, shiny with very few longitudinal ridge. Mandibles: Smooth, shiny. Anterior margin of clypeus: Evenly convex. Hairs: Hairs erect allover except adpressed on the antennae. Postocular distance vs. cephalic length (POC/CL): 0.372. Eye vs. absolute cephalic size (EYE/CS): 0.265. Frontal carina distance vs. absolute cephalic size (FR/CS): 0.420. Scape length vs. absolute cephalic size (SL/CL): 0.723.

Alitrunk.

Color and color pattern: Dark brown. Sculpture: Dorsal region of mesosoma rugoso-reticulate with areolate ground sculpture. Lateral region of pronotum rugoso-reticulate with areolate ground sculpture. Mesopleuron a rugoso-reticulate with areolate ground sculpture. Metapleuron rugoso-reticulate with areolate ground sculpture. Propodeal spines: Short, pointed, triangulate. Hairs: Erect hairs mainly on dorsal face of pronotum, mesonotum and propodeum. Mesosoma length vs. absolute cephalic size (ML/CS): 1.292. Maximum mesosoma width vs. absolute cephalic size (MW/CS): 0.695. Spine length vs. absolute cephalic size (SPST/CS): 0.280. Minimum spine distance at its base vs. absolute cephalic size (SPBA/CS): 0.302. Maximum spine distance at its tip vs. absolute cephalic size (SPWI/CS): 0.290. Apical spine distance vs. absolute cephalic size (SPTI/CS): 0.258.

Pedicel.

Color: Dark brown. Sculpture: Petiole and postpetiole nodes rugoso-reticulate with areolate ground sculpture, the ventral parts smooth. Petiole node: In profile, dorsal face of petiole node curving backwards without angle. Petiole ventral lobe/Ventral projection (lamella): In profile, posteroventral part of petiole forms straight, slightly concave line. Lamella slightly pointed. Ventral postpetiolar process/Inferior tooth: Rounded. Hair: Erect hairs on petiole, postpetiole nodes and ventral pp process. Petiole width vs. Postpetiole width (PEW/PPW): 0.659.

Gaster.

Color: Dark brown. Hairs: Erect hairs allover the gaster. Sculpture: Smooth, shiny.

Diagnosis in key.

Taxonomic changes

Formerly considered junior synonym of this *T*. *microcellatus* (Soudek, 1925) species is revived from synonymy. For details see description of the latter species.

Geographic distribution

The species is known only from the type locality near Kokshetau, Kazakhstan (Fig 15) [55].

Host ant usage

Specimens of the host species belonging to the type specimen were not available to us, but based on the literature the host species is *Temnothorax serviculus* (Ruzsky, 1902) [55].

### *Temnothorax kraussei* (Emery, 1915)

*Epimyrma kraussei* Emery [18]: 262 (w.q.) ITALY (Sardinia).

Combination in *Myrmoxenus*: Cagniant [48]: 198; Bračko [55]: 139

Combination in *Temnothorax*: Ward et al. [29]: 75.

Senior synonym of *Temnothorax vandeli* (Santschi, 1927): Buschinger et al. [44]: 274

Senior synonym of *Temnothorax foreli* Menozzi, 1921: Buschinger et al. [44]: 274

Type material investigated.

One *Temnothorax kraussei* syntype gyne was investigated from type locality: Italy: Sardinia, Sorgono, 14.III.1913, leg. A. Krausse (1g-MSNG); One syntype worker and one syntype gyne of *Epimyrma kraussei* were investigated from type locality: Italy: Calabria, vic. Sambiase (no collector’s name, perhaps F. Silvestri), (1w,1g-NHMB, CASENT0912886, CASENT0912887); One holotype gyne of *Epimyrma vandeli* was investigated from the type locality: France: Tarn-et-Garonne, Miramont-de-Quercy, 24.IX.1926, leg. A. Vandel, (1g-NHMB).

**Fig 19:**
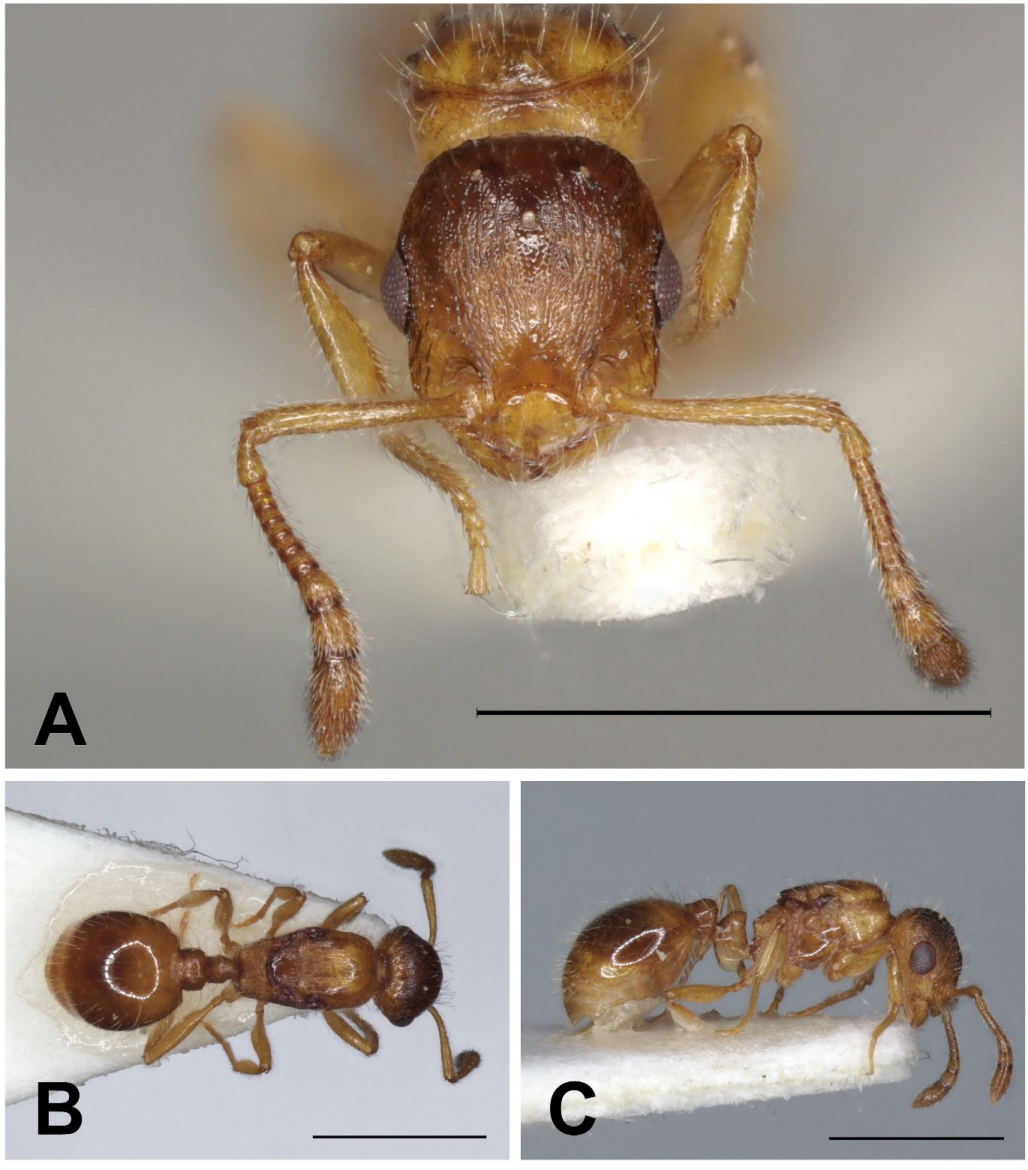
*Temnothorax kraussei* gynes. Head in full-face view (A), dorsal view of the body (B), lateral view of the body (C), scale bar: 1 mm.

Description of gynes (Fig 19, Table 4).

Body color brown, pale brown. Body color pattern concolorous.

Head.

Absolute cephalic size: 624±21.793 [569, 674]. Cephalic length vs. maximum width of head capsule (CL/CWb): 1.213±0.024 [1.148, 1.280]. Color: Brown. Frons: Brown. Vertex: Brown. Scape: Pale brown. Antennal club: Brown. Head frontal sculpture: Rugoso-reticulate with areolate ground sculpture. Clypeus: Smooth, shiny with well-developed longitudinal ridges. Mandibles: Smooth, at and t2 long, t3-4 small rounded, bt small rounded. Anterior margin of clypeus: Evenly convex, slightly pointed. Hairs: Hairs erect allover except adpressed on the antennae. Postocular distance vs. cephalic length (POC/CL): 0.353±0.008 [0.332, 0.375]. Eye vs. absolute cephalic size (EYE/CS): 0.271±0.009 [0.216, 0.293]. Frontal carina distance vs. absolute cephalic size (FR/CS): 0.336±0.010 [0.307, 0.365]. Scape length vs. absolute cephalic size (SL/CL): 0.687±0.019 [0.635, 0.733].

Alitrunk.

Color and color pattern: Brown, pale brown. Mesoscutellum often dark brown. Sculpture: Dorsal region of mesosoma extensively smooth, feeble areolate with parallel costulate main sculpture. Lateral region of pronotum rugoso-areolate. Anepisternum feeble areolate, shiny. Katepisternum smooth, shiny, upper third areolate. Metapleuron rugoso-areolate. Propodeal spines: Very short, slighlty rounded, triangulate. Hairs: Erect hairs, mainly on the dorsal side of pronotum, mesonotum. Mesosoma length vs. absolute cephalic size (ML/CS): 1.421±0.039 [1.162, 1.490]. Maximum mesosoma width vs. absolute cephalic size (MW/CS): 0.809±0.028 [0.711, 0.893]. Spine length vs. absolute cephalic size (SPST/CS): 0.251±0.013 [0.212, 0.287]. Minimum spine distance at its base vs. absolute cephalic size (SPBA/CS): NA. Maximum spine distance at its tip vs. absolute cephalic size (SPWI/CS): 0.389±0.020 [0.323, 0.443]. Apical spine distance vs. absolute cephalic size (SPTI/CS): 0.359±0.019 [0.281, 0.399].

Pedicel.

Color: Brown, pale brown. Sculpture: Petiole and postpetiole nodes feeble areolate, the ventral parts smooth. Petiole node: In profile, dorsal face of petiole node slightly pointed anteriorly, curving backwards with or without angle. Petiole ventral lobe/Ventral projection (lamella): In profile, posteroventral part of petiole forms convex or sometimes partly straight (but never concave) line. Ventral postpetiolar process/Inferior tooth: Slightly rounded. Hairs: Erect hairs on petiole, postpetiole nodes and ventral pp process. Petiole width vs. postpetiole width (PEW/PPW): 0.605±0.025 [0.587, 0.753].

Gaster.

Color: Brown, dark brown. Hairs: Erect hairs allover the gaster. Sculpture: Smooth, shiny.

**Fig 20:**
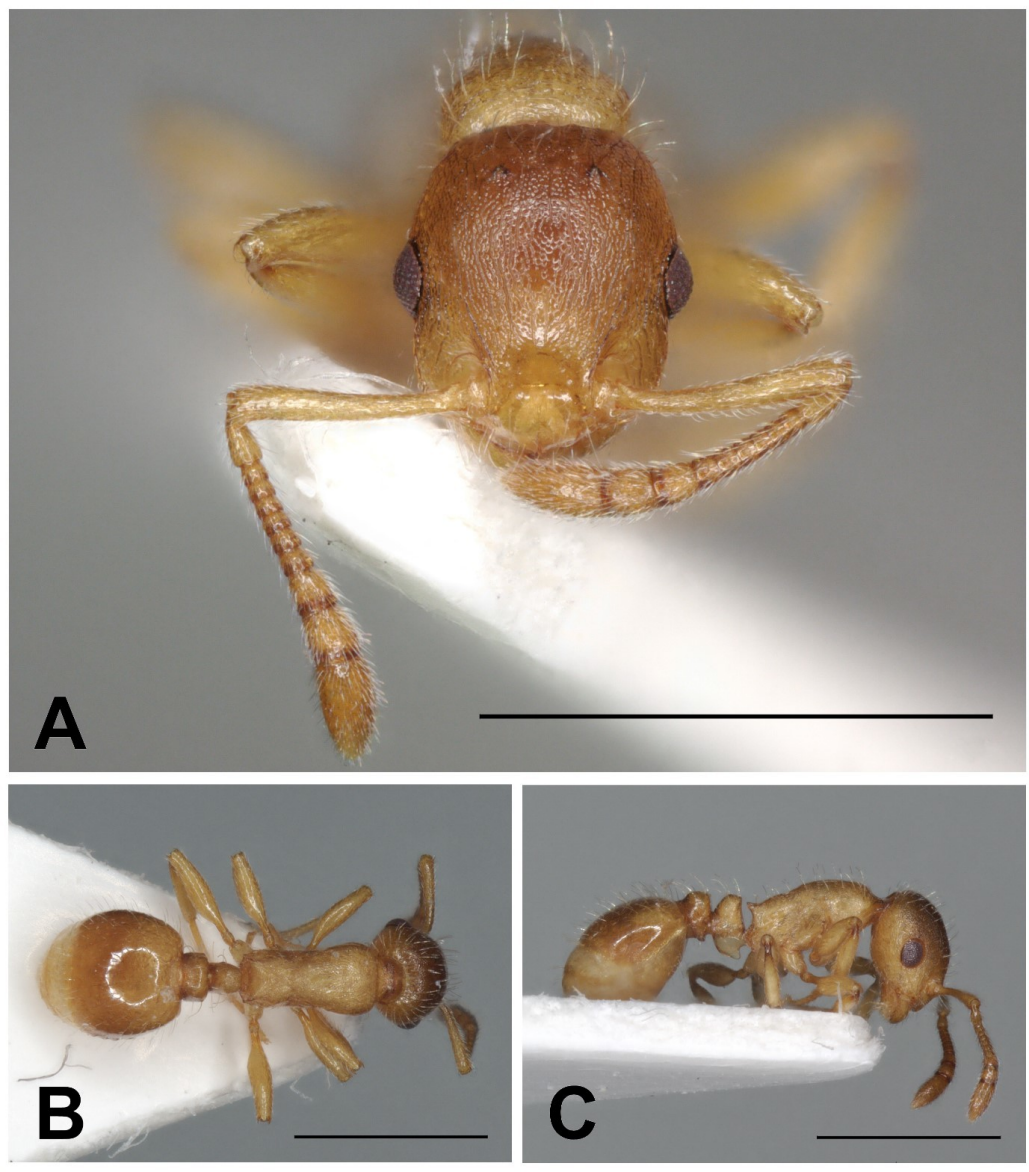
*Temnothorax kraussei* worker. Head in full-face view (A), dorsal view of the body (B), lateral view of the body (C), scale bar: 1 mm.

Description of workers (Fig 20 and Table 5).

Body color brown, pale brown. Body color pattern concolorous.

Head.

Absolute cephalic size: 599±19.488 [561, 650]. Cephalic length vs. maximum width of head capsule (CL/CWb): 1.268±0.022 [1.220, 1.319]. Color: Brown, pale brown. Frons: Brown, pale brown. Vertex: Brown. Scape: Pale brown. Antennal club: Pale brown, brown. Head frontal sculpture: Rugoso-reticulate with areolate ground sculpture. Clypeus: Smooth, shiny with very few longitudinal ridge. Mandibles: smooth, shiny, 4–5 teeth, at long, t2 moderate, t3-4 short or reducated, bt short. Anterior margin of clypeus: Evenly convex, pointed. Hairs: Hairs erect allover except adpressed on the antennae. Postocular distance vs. cephalic length (POC/CL): 0.354±0.009 [0.330, 0.371]. Eye vs. absolute cephalic size (EYE/CS): 0.236±0.008 [0.217, 0.250]. Frontal carina distance vs. absolute cephalic size (FR/CS): 0.337±0.009 [0.316, 0.362]. Scape length vs. absolute cephalic size (SL/CL): 0.706±0.018 [0.670, 0.748].

Alitrunk.

Color and color pattern: Pale brown. Sculpture: Dorsal region of mesosoma rugulose with areolate ground sculpture. Lateral region of pronotum areolate ground sculpture, main sculpture forked costate. Mesopleuron areolate ground sculpture superimposed by dispersed rugulae. Metapleuron areolate ground sculpture superimposed by dispersed rugulae. Propodeal spines: Medium, pointed, triangulate. Hairs: Long erect hairs mainly on dorsal face of pronotum, mesonotum and propodeum. Mesosoma length vs. absolute cephalic size (ML/CS): 1.210±0.034 [1.154, 1.323]. Maximum mesosoma width vs. absolute cephalic size (MW/CS): 0.623±0.016 [0.592, 0.671]. Spine length vs. absolute cephalic size (SPST/CS): 0.256±0.018 [0.210, 0.301]. Minimum spine distance at its base vs. absolute cephalic size (SPBA/CS): 0.348±0.017 [0.311, 0.407]. Maximum spine distance at its tip vs. absolute cephalic size (SPWI/CS): 0.381±0.025 [0.326, 0.440]. Apical spine distance vs. absolute cephalic size (SPTI/CS): 0.365±0.024 [0.313, 0.427].

Pedicel.

Color: Pale brown. Sculpture: Petiole and postpetiole feeble areolate or smooth, the ventral parts smooth. Petiole node: In profile, dorsal face of petiole node curving backwards without angle. Petiole ventral lobe/Ventral projection (lamella): In side view, posteroventral part of petiole forms convex or sometimes partly straight or slightly concave line. Ventral postpetiolar process/Inferior tooth: Slightly rounded. Hairs: Erect hairs on petiole, postpetiole nodes and ventral pp process. Petiole width vs. Postpetiole width (PEW/PPW): 0.653±0.026 [0.612, 0.740].

Gaster.

Color: Brown, pale brown. Hairs: Erect hairs all over the gaster.

Diagnosis in key. Further details can be found under description of *T*. *algerianus*.

Geographic distribution

The species is widespread in the Mediterranean. It is found in North Africa in Algeria, and in most of the northern Mediterranean: Spain, France, Corsica, Sardinia, Italy, Croatia, Bulgaria and Türkiye (Fig 7). Furthermore, it is known from Morocco, Tunisia, Portugal, Slovenia, Serbia and Greece [44, 46].

Host ant usage

Based on the 60 samples we examined, the only known host species is *Temnothorax recedens* (no host specimens were available in 15 cases) and no other host species is mentioned in the literature [31, 44].

### *Temnothorax microcellatus* (Soudek, 1925) revived from synonymy

Combination in *Epimyrma*: Bolton [43]: 188.

Combination in *Myrmoxenus*: Schulz & Sanetra [25]: 159.

Combination in *Temnothorax*: Ward et al. [29]: 75.

*Temnothorax gordiagini menozzii* **syn. n.**

Type material investigated.

One *Myrmetareus microcellatus* syntype gyne and one synytpe worker was investigated from type locality: Croatia: Dalmatia, Gulf of Kotor (Cattaro), Savina Monastery, nr Erceg Novi (Castelnuovo), VII.1923, leg. S. Soudek (1g-NHMP, 1w-NHMP). One *Myrmoxenus gordiagini subsp*. *menozzii* syntype gyne was investigated from the type locality: Italy: Momiano (Istria settentr.), 7.IX.1920, leg. B. Finzi (1g-MCZC).

**Fig 21:**
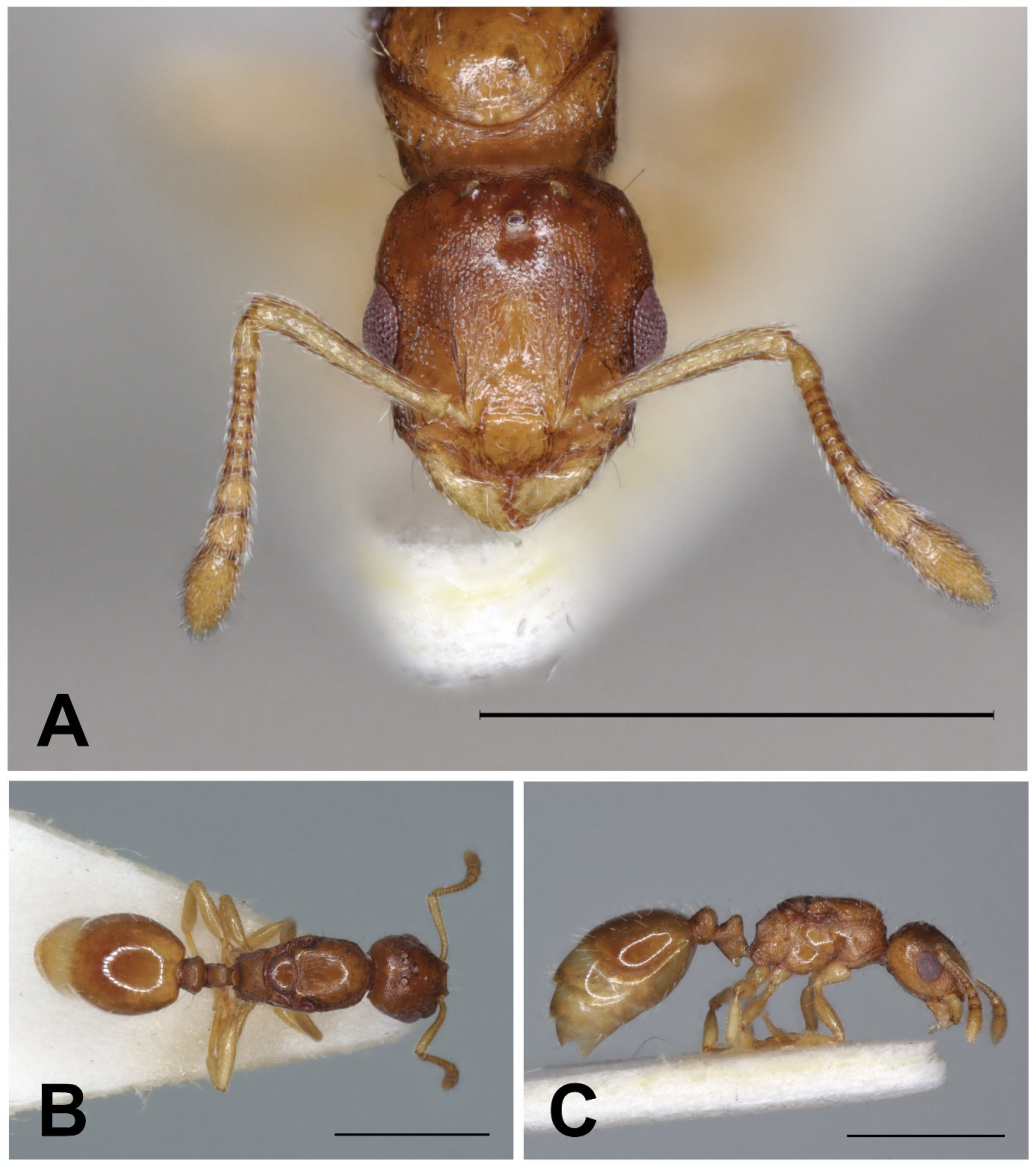
*Temnothorax microcellatus* gyne. Head in full-face view (A), dorsal view of the body (B), lateral view of the body (C), scale bar: 1 mm.

Description of gynes (Fig 21 and Table 4).

Body color dark brown, almost black. Body color pattern concolorous.

Head.

Absolute cephalic size: 605±15.596 [569, 639]. Cephalic length vs. maximum width of head capsule (CL/CWb): 1.127±0.021 [1.080, 1.198]. Color: Dark brown. Frons: Dark brown. Vertex: Dark brown. Scape: Pale brown. Antennal club: Pale brown, brown. Head frontal sculpture: Rugoso-reticulate with areolate ground sculpture. Clypeus: Smooth, shiny with well-developed longitudinal ridges. Mandibles: Smooth, bt rounded, t3-4 similar in size, roundes, t2 bigger than t3-4, at long, pointed. Anterior margin of clypeus: Evenly convex. Hairs: Hairs erect allover except adpressed on the antennae. Postocular distance vs. cephalic length (POC/CL): 0.365±0.009 [0.348, 0.384]. Eye vs. absolute cephalic size (EYE/CS): 0.313±0.009 [0.288, 0.339]. Frontal carina distance vs. absolute cephalic size (FR/CS): 0.353±0.009 [0.335, 0.370]. Scape length vs. absolute cephalic size (SL/CL): 0.714±0.019 [0.666, 0.761].

Alitrunk.

Color and color pattern: Uniformly dark brown, without any noticeable pattern. Sculpture: Dorsal region of mesosoma rugoso-reticulate with aerolate ground sculpture. Lateral region of pronotum rugoso-reticulate with areolate ground sculpture. Anepisternum smooth, shiny, upper third rugoso-reticulate. Katepisternum smooth, shiny. Metapleuron rugoso-reticulate with areolate ground scupture. Propodeal spines: Very short, slighlty pointed, triangulate. Hairs: Erect hairs, mainly on the dorsal side of pronotum, mesonotum and on the posterior face of propodeum. Mesosoma length vs. absolute cephalic size (ML/CS): 1.473±0.036 [1.391, 1.578]. Maximum mesosoma width vs. absolute cephalic size (MW/CS): 0.853±0.042 [0.781, 0.946]. Spine length vs. absolute cephalic size (SPST/CS): 0.249±0.009 [0.224, 0.264]. Minimum spine distance at its base vs. absolute cephalic size (SPBA/CS): NA. Maximum spine distance at its tip vs. absolute cephalic size (SPWI/CS): 0.378±0.015 [0.338, 0.421]. Apical spine distance vs. absolute cephalic size (SPTI/CS): 0.325±0.013 [0.301, 0.359].

Pedicel.

Color: Dark brown. Sculpture: Petiole and postpetiole nodes reticulated, the ventral parts smooth. Petiole node: In profile, dorsal face of petiole node curving backwards without angle. Petiole ventral lobe/Ventral projection (lamella): In profile, posteroventral part of petiole forms straight (but never concave) line. Lamella pointed. Ventral postpetiolar process/Inferior tooth: Strongly rounded. Hairs: Erect hairs on petiole, postpetiole nodes and ventral pp process. Petiole width vs. Postpetiole width (PEW/PPW): 0.640±0.027 [0.578, 0.689].

Gaster.

Color: Dark brown. Hairs: Erect hairs allover the gaster. Sculpture: Smooth, shiny.

**Fig 22:**
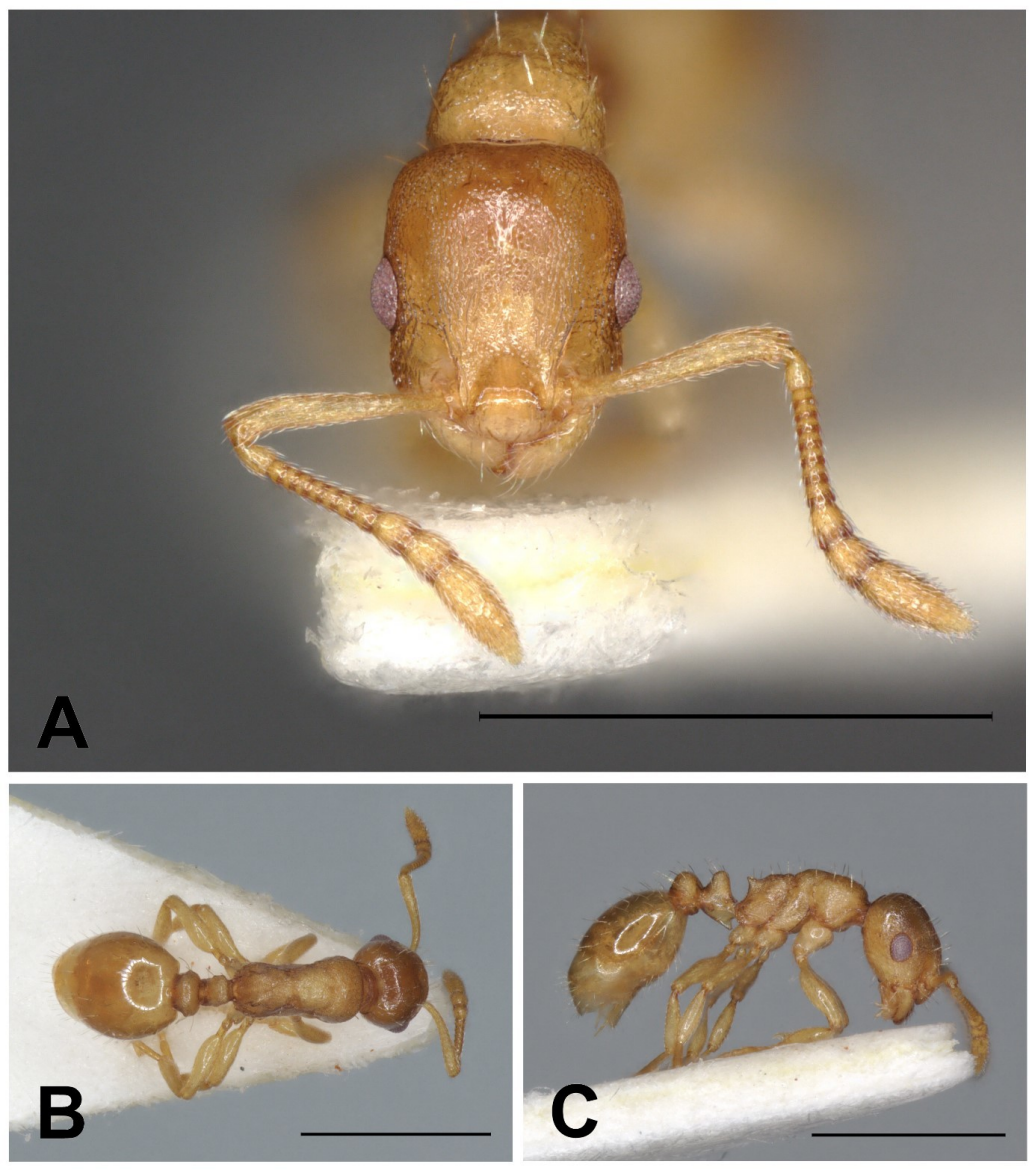
*Temnothorax microcellatus* worker. Head in full-face view (A), dorsal view of the body (B), lateral view of the body (C), scale bar: 1 mm.

Description of workers (Fig 22 and Table 5).

Body color brown, pale brown. Body color pattern concolorous.

Head.

Absolute cephalic size: 590±20.033 [546, 654]. Cephalic length vs. maximum width of head capsule (CL/CWb): 1.192±0.020 [1.144, 1.230]. Color: Brown, pale brown. Frons: Brown, pale brown. Vertex: Brown, pale brown. Scape: Brown, pale brown. Antennal club: Brown, pale brown. Head frontal sculpture: Reticulate with areolate ground sculpture. Moderately shiny. Clypeus: Smooth, shiny with a few longitudinal ridges. Mandibles: Smooth, shiny. Anterior margin of clypeus: Evenly convex. Hairs: Hairs erect allover except adpressed on the antennae. Postocular distance vs. cephalic length (POC/CL): 0.369±0.019 [0.348, 0.493]. Eye vs. absolute cephalic size (EYE/CS): 0.258±0.010 [0.237, 0.283]. Frontal carina distance vs. absolute cephalic size (FR/CS): 0.361±0.008 [0.337, 0.374]. Scape length vs. absolute cephalic size (SL/CL): 0.734±0.018 [0.699, 0.783].

Alitrunk.

Color and color pattern: Brown, Pale brown. Sculpture: Dorsal region of mesosoma ruguloso-reticulate with areolate ground sculpture. Lateral region of pronotum areolate ground sculpture, main sculpture reticulate. Mesopleuron areolate. Metapleuron areolate ground sculpture superimposed by dispersed rugulae. Propodeal spines: Short, pointed, triangulate. Hairs: Erect hairs mainly on dorsal face of pronotum, mesonotum and propodeum. Mesosoma length vs. absolute cephalic size (ML/CS): 1.251±0.026 [1.203, 1.300]. Maximum mesosoma width vs. absolute cephalic size (MW/CS): 0.663±0.012 [0.642, 0.705]. Spine length vs. absolute cephalic size (SPST/CS): 0.227±0.014 [0.190, 0.266]. Minimum spine distance at its base vs. absolute cephalic size (SPBA/CS): 0.312±0.016 [0.285, 0.369]. Maximum spine distance at its tip vs. absolute cephalic size (SPWI/CS): 0.328±0.015 [0.296, 0.373]. Apical spine distance vs. absolute cephalic size (SPTI/CS): 0.308±0.016 [0.277, 0.354].

Pedicel.

**Fig 23:**
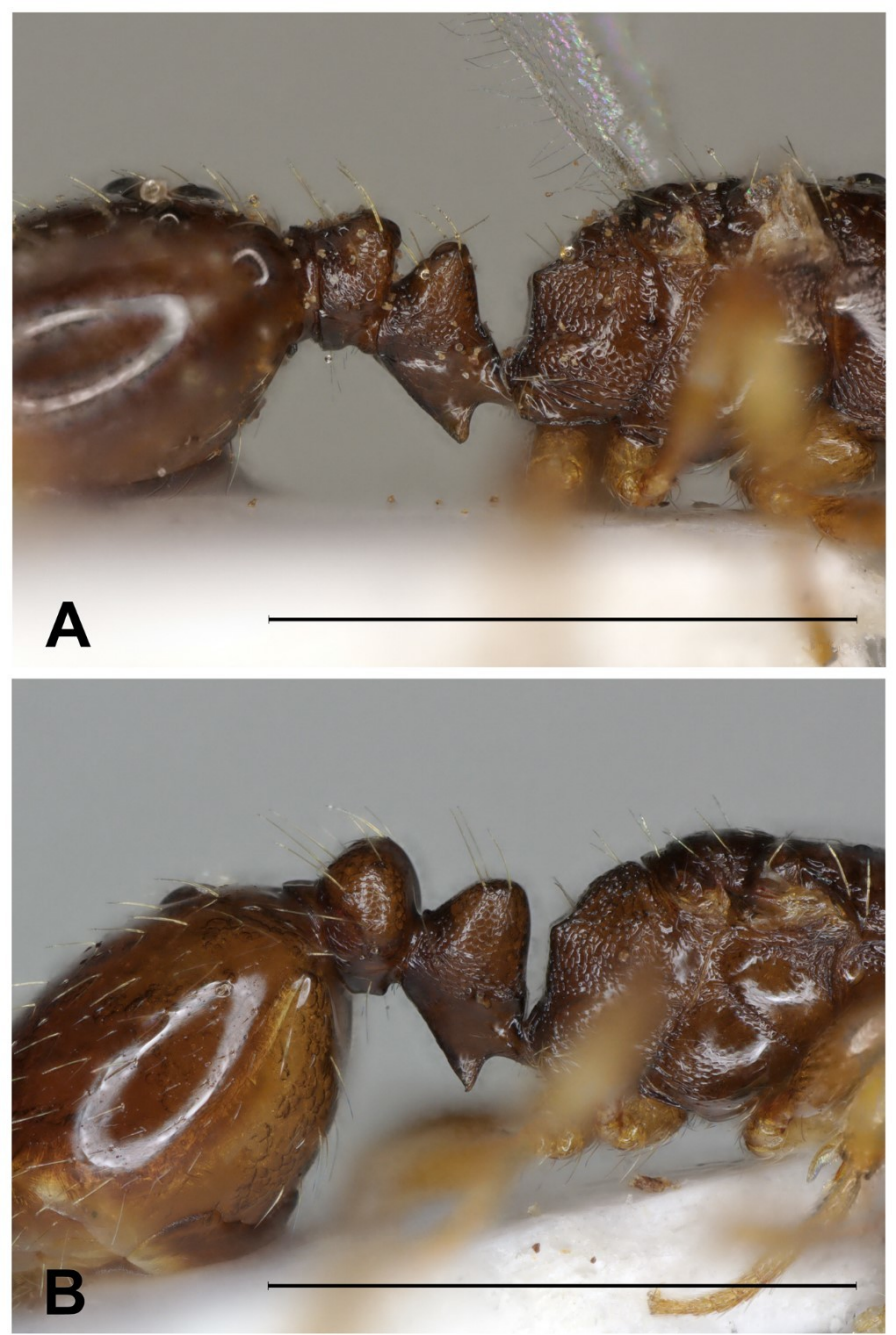
The petiole of *T*. *microcellatus* individuals can be highly variable. Typical appearance of the petiole (A). Atypical variant of the petiole (B), scale bar: 1 mm.

Color: Brown, pale brown. Sculpture: Petiole and postpetiole areolate, the ventral parts smooth. Petiole node: In profile, dorsal face of petiole node curving backwards without angle. Petiole ventral lobe/Ventral projection (lamella): In profile, posteroventral part of petiole forms straight, sometimes slightly concave line (Fig 23). Lamella slightly pointed. Ventral postpetiolar process/Inferior tooth: Rounded. Hairs: Erect hairs on petiole, postpetiole nodes and ventral pp process. Petiole width vs. Postpetiole width (PEW/PPW): 0.655±0.030 [0.592, 0.716].

Gaster.

Color: Brown, Pale brown. Hairs: Erect hairs all over the gaster. Sculpture: Smooth, shiny.

Diagnosis in key.

Taxonomic changes

**Fig 24:**
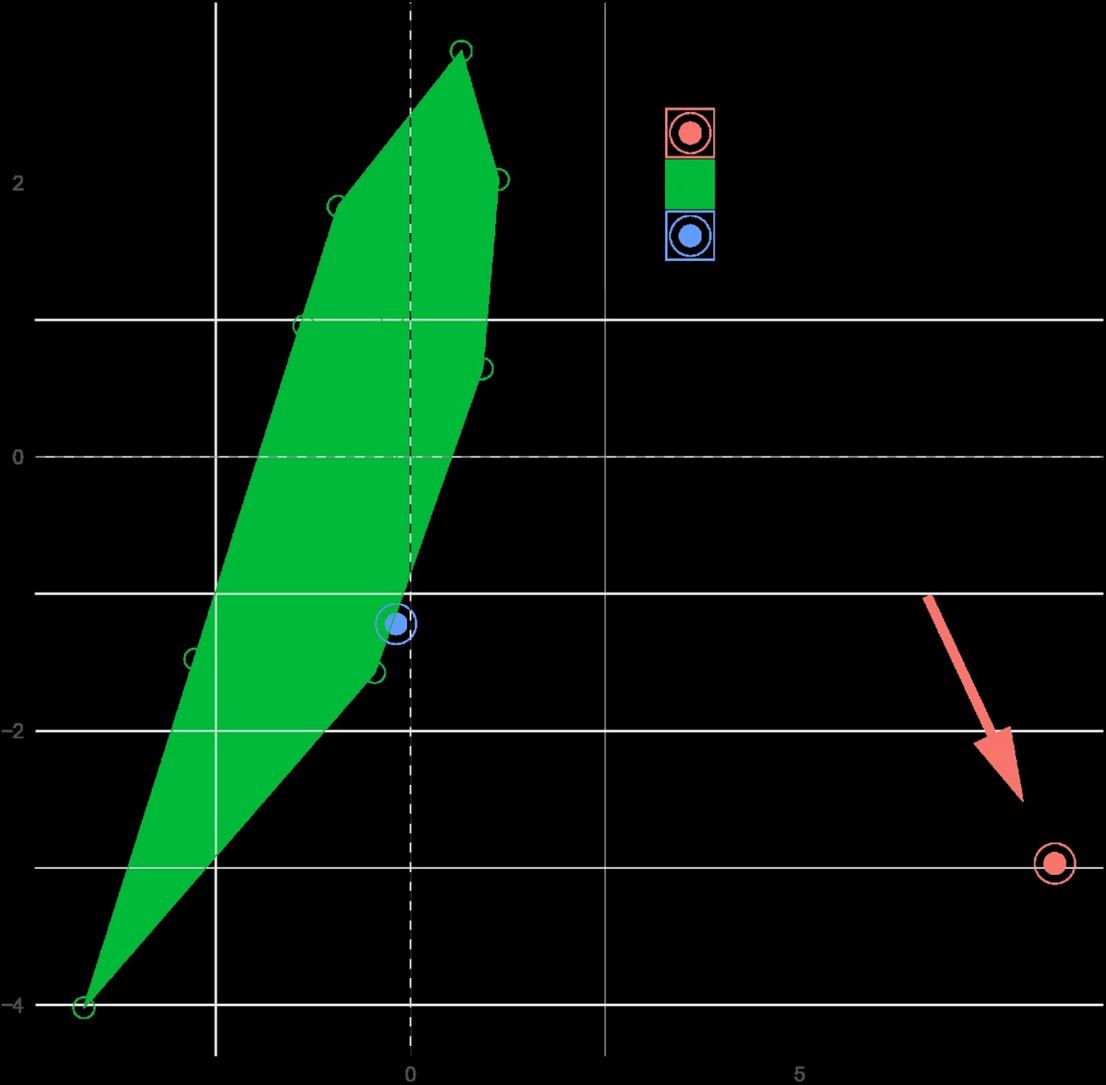
PCA plot of morphometric data of *Temnothorax microcellatus* (green) and the relevant type material are illustrated on two principal components (Dim 1, Dim 2). The syntype worker of *T*. *microcellatus* (blue) is nested in the *microcellatus* cluster and *T*. *gordiagini* (red) takes a peripheral position.

The taxon *Temnothorax* (*Myrmoxenus*) *gordiagini* (Ruzsky, 1902) had been introduced in the European ant fauna, as a senior syonym of *T*. *microcellatus*. However, the current morphological analyses reveal that the type specimen of *T*. *gordiagini* (a worker individual) is not nested in the cluster of *T*. *microcellatus* workers from the East Mediterranean basin (Fig 24) but appears an outlier far beyond the range of the distribution of *T*. *microcellatus* (Fig 15). Therefore, we propose reviving *T*. *microcellatus* (Soudek, 1925) from synonymy.

Geographic Distribution

The species is distributed from northern Italy through Croatia, Montenegro, Bulgaria, Greece to central Türkiye (Fig 15) and known from Bosnia and Herzegovina, Serbia, Macedonia and Slovenia [20, 56, 57].

Host ant usage

Out of the 22 samples we examined, *Temnothorax lichtensteini* (Bondroit, 1918) was the host species in 16 cases, *T*. *nylanderi* (Foerster, 1850) in 1 case and *T*. *unifasciatus* (Latreille, 1798) in 1 case, while in 4 cases no specimens of the host species were available. Host species mentioned in the literature are *T*. *lichtensteini*, *T*. *nylanderi*, *T*. *unifasciatus*, *T*. *graecus* (Forel, 1911), *T*. *korbi* (Emery, 1924), *T*. *bulgaricus* (Forel, 1892) [20, 56, 57].

### *Temnothorax ravouxi* (André, 1896)

*Formicoxenus ravouxi* André [58]: 367 (q.) FRANCE.

Combination in *Epimyrma*: Emery [18]: 262.

Combination in *Myrmoxenus*: Schulz & Sanetra [25]: 162.

Combination in *Temnothorax*: Ward et al. [29]: 75.

Senior synonym of *Temnothorax goesswaldi*: Buschinger [45]: 352.

Senior synonym of *Temnothorax zaleskyi*: Báthori et al. [16]: 4.

Senior synonym of *Temnothorax tamarae* **syn. n.**

Investigated type material.

Four syntype gynes of *Myrmoxenus ravouxi* were investigated from the type locality: France: Nyons (Drôme), leg. Ravoux, (4g-MNHN). Two syntype workers and one syntype gyne of *Epimyrma goesswaldi* were investigated from the type locality: Germany: Lindelbach, Würzburg, leg. K. Gösswald, (2w,1q-NHMB, CASENT0912888). Four gynes and one worker of *Epimyrma zaleskyi* were investigated from type locality: Slovakia: Babalou in Dolním Almáši, between Levice and Banska Štiavnik, 19.ix.1938, leg. M. Záleský (These specimens were not labeled as types, but presumably this set was used for the original description by J. Sadil).

**Fig 25:**
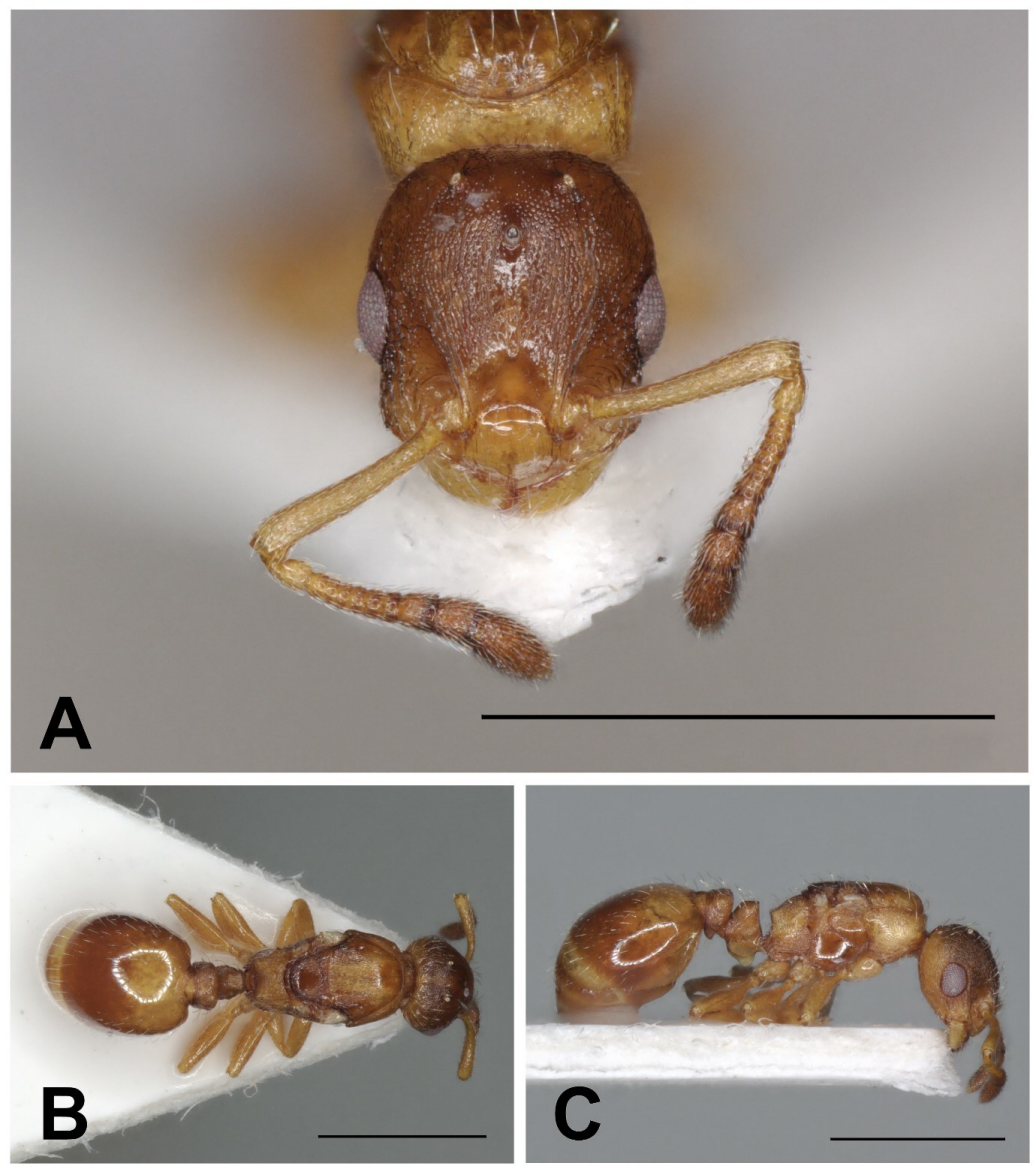
*Temnothorax ravouxi* gyne. Head in full-face view (A), dorsal view of the body (B), lateral view of the body (C), scale bar: 1 mm.

Descriptions of gynes (Fig 25 and Table 4).

Body color brown, pale brown. Body color pattern concolorous.

Head.

Absolute cephalic size: 635±20.777 [591, 675]. Cephalic length vs. maximum width of head capsule (CL/CWb): 1.181±0.025 [1.135, 1.250]. Color: Brown. Frons: Brown. Vertex: Brown. Scape: Pale brown. Antennal club: Brown. Head frontal sculpture: Rugoso-reticulate with areolate ground sculpture. Moderately shiny. Clypeus: Smooth, shiny with well-developed longitudinal ridges. Mandibles: Smooth, 4–5 teeth, bt, t2-t3 short, reducated, at, t1 long. Anterior margin of clypeus: evenly convex, slightly pointed. Hairs: Hairs erect allover except adpressed on the antennae. Postocular distance vs. cephalic length (POC/CL): 0.362±0.011 [0.327, 0.411]. Eye vs. absolute cephalic size (EYE/CS): 0.292±0.009 [0.265, 0.311]. Frontal carina distance vs. absolute cephalic size (FR/CS): 0.357±0.015 [0.324, 0.395]. Scape length vs. absolute cephalic size (SL/CL): 0.651±0.017 [0.610, 0.699].

Alitrunk.

Color and color pattern: Brown, pale brown, Mesoscutellum often darker. Sculpture: Dorsal region of mesosoma rugulose with rugoso-areolate ground sculpture, sometimes with parallel costulate main sculpture. Lateral region of pronotum rugoso-areolate. Anepisternum areolate lower third smooth, shiny. Katepisternum smooth, shiny, upper third areolate. Metapleuron rugoso-areolate. Propodeal spines: Short, slightly pointed, triangulate. Hairs: Erect hairs, mainly on the dorsal side of pronotum, mesonotum and on the posterior face of propodeum. Mesosoma length vs. absolute cephalic size (ML/CS): 1.517±0.034 [1.408, 1.589]. Maximum mesosoma width vs. absolute cephalic size (MW/CS): 0.890±0.030 [0.806, 0.967]. Spine length vs. absolute cephalic size (SPST/CS): 0.266±0.013 [0.230, 0.304]. Minimum spine distance at its base vs. absolute cephalic size (SPBA/CS): NA. Maximum spine distance at its tip vs. absolute cephalic size (SPWI/CS): 0.410±0.020 [0.361, 0.463]. Apical spine distance vs. absolute cephalic size (SPTI/CS): 0.368±0.018 [0.330, 0.409].

Pedicel.

Color: Brown, Pale brown. Sculpture: Petiole and postpetiole nodes feeble areolate, the ventral parts smooth. Petiole node: In profile, dorsal face of petiole node curving backwards with or without angle. Petiole ventral lobe/Ventral projection (lamella): In profile, posteroventral part of petiole forms convex or sometimes partly straight or slightly concave line. Ventral postpetiolar process/Inferior tooth: Slightly pointed. Hairs: Erect hairs on petiole, postpetiole nodes and ventral pp process. Petiole width vs. Postpetiole width (PEW/PPW): 0.672±0.029 [0.566, 0.748].

Gaster.

Color: Brown, first tergite usually pale brown at base. Hairs: Erect hairs allover the gaster. Sculpture: Smooth, shiny.

**Fig 26:**
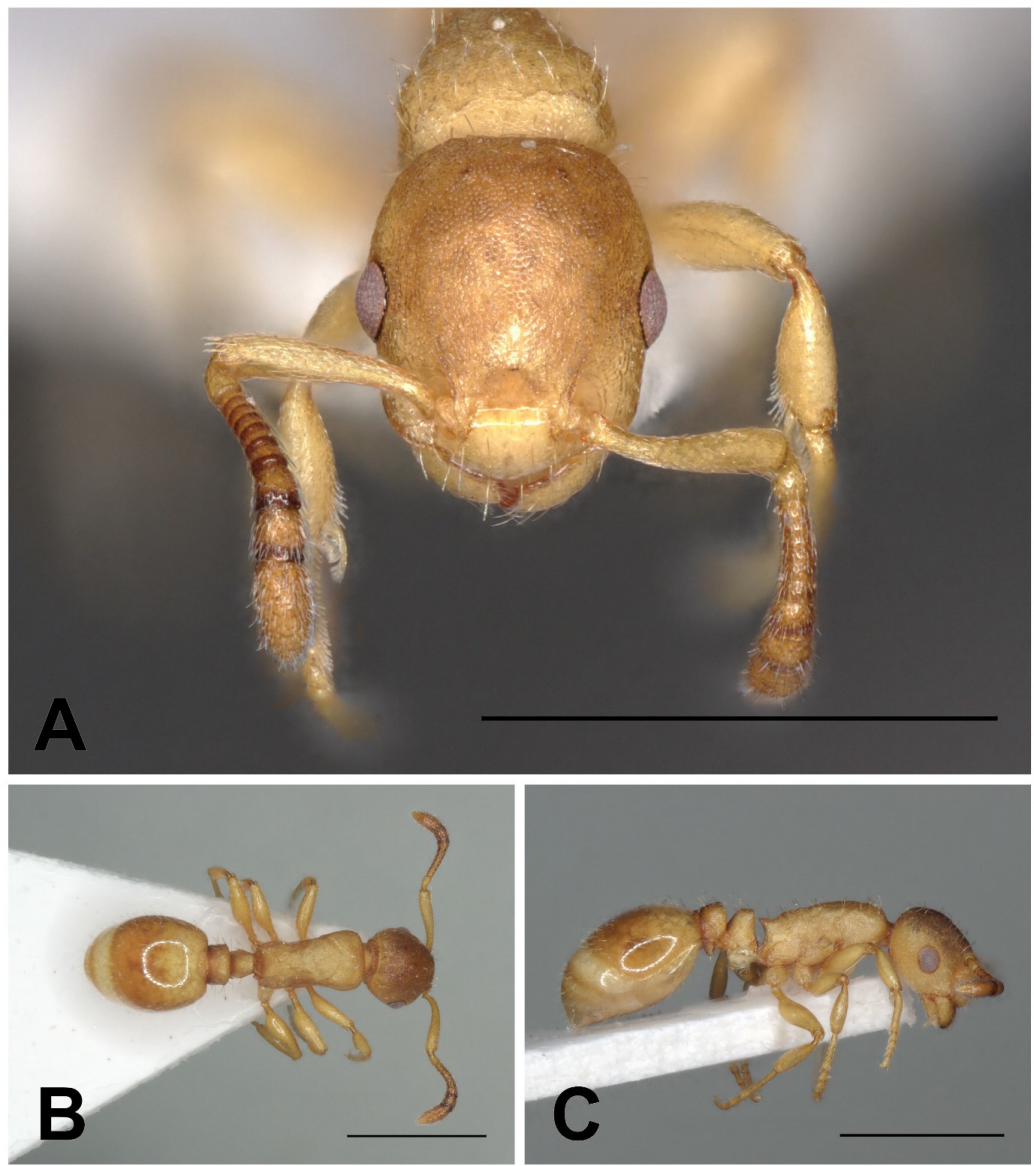
*Temnothorax ravouxi* worker. Head in full-face view (A), dorsal view of the body (B), lateral view of the body (C), scale bar: 1 mm.

Description of workers (Fig 26 and Table 5).

Body color brown, pale brown. Body color pattern concolorous.

Head.

Absolute cephalic size: 614±26.021 [551, 665]. Cephalic length vs. maximum width of head capsule (CL/CWb): 1.232±0.027 [1.155, 1.323]. Color: Brown, pale brown. Frons: Brown, pale brown. Vertex: Brown. Scape: Pale brown. Antennal club: Brown. Head frontal sculpture: Rugoso-reticulate with areolate ground sculpture. Moderately shiny. Clypeus: Smooth, shiny with few longitudinal ridge. Mandibles: Smooth, shiny, 4–5 teeth, at long, t2 moderate, t3-4 short rounded, bt short rounded. Anterior margin of clypeus: Evenly convex, slightly pointed. Hairs: Hairs erect allover except adpressed on the antennae. Postocular distance vs. cephalic length (POC/CL): 0.368±0.010 [0.342, 0.407]. Eye vs. absolute cephalic size (EYE/CS): 0.250±0.009 [0.226, 0.280]. Frontal carina distance vs. absolute cephalic size (FR/CS): 0.362±0.014 [0.328, 0.406]. Scape length vs. absolute cephalic size (SL/CL): 0.672±0.022 [0.613, 0.733].

Alitrunk.

Color and color pattern: Pale brown. Sculpture: Dorsal region of mesosoma ruguso-reticulate with areolate ground sculpture. Lateral region of pronotum areolate ground sculpture, main sculpture reticulate. Mesopleuron areolate. Metapleuron areolate ground sculpture superimposed by dispersed rugulae. Propodeal spines: Short, pointed, triangulate. Hairs: Erect hairs mainly on dorsal face of pronotum, mesonotum and propodeum. Mesosoma length vs. absolute cephalic size (ML/CS): 1.274±0.037 [1.160, 1.370]. Maximum mesosoma width vs. absolute cephalic size (MW/CS): 0.672±0.022 [0.619, 0.731]. Spine length vs. absolute cephalic size (SPST/CS): 0.262±0.021 [0.215, 0.316]. Minimum spine distance at its base vs. absolute cephalic size (SPBA/CS): 0.356±0.021 [0.296, 0.405]. Maximum spine distance at its tip vs. absolute cephalic size (SPWI/CS): 0.378±0.026 [0.309, 0.459]. Apical spine distance vs. absolute cephalic size (SPTI/CS): 0.357±0.027 [0.278, 0.435].

Pedicel.

**Fig 27:**
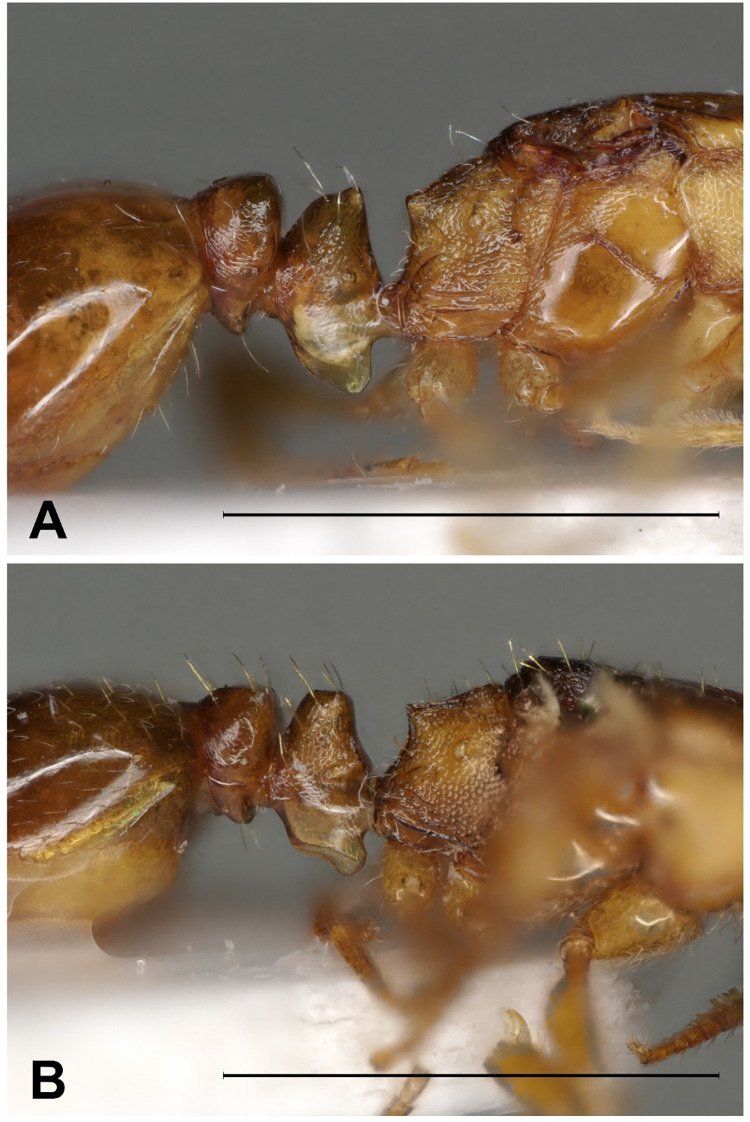
The petiole of *T*. *ravouxi* individuals can be highly variable. Typical appearance of the petiole (A). Atypical variant of the petiole (B), scale bar: 1 mm.

Color: Pale brown. Sculpture: Petiole and postpetiole slightly reticulate, the ventral parts smooth. Petiole node: In profile, dorsal face of petiole node curving backwards without angle. Petiole ventral lobe/Ventral projection (lamella): In profile, posteroventral part of petiole forms slightly concave or partly straight line (Fig 27). Ventral postpetiolar process/Inferior tooth: Slightly pointed. Hairs: Erect hairs on petiole, postpetiole nodes and ventral pp process. Petiole width vs. Postpetiole width (PEW/PPW): 0.686±0.028 [0.609, 0.766].

Gaster.

Color: Brown, pale brown. Hairs: Erect hairs allover the gaster. Sculpture: Smooth, shiny.

Geographic distribution

The species is widespread in the Western Palaearctic, found in Algeria in North Africa, and in Spain, France, Italy, Switzerland, Germany, Slovakia, Hungary, Albania, Türkiye, Georgia and Russia. It is also known from Austria, Czech Republic, Corsica, Sardinia, Poland, Serbia, Ukraine, Bulgaria and Greece [46].

Host ant usage

Of the 76 samples we examined, *Temnothorax unifasciatus* was the host species in 40 cases, *T*. *affinis* (Mayr, 1855) in 12 cases, *T*. *nigriceps* (Mayr, 1855) in 4 cases and *T*. *lichtensteini* (new host species) in 1 case, and no specimens of the host species were available for 19 samples. Other host species mentioned in the literature and known from unpublished data: *T*. *albipennis* (Curtis, 1854), *T*. *corticalis* (Schenck, 1852), *T*. *jailensis* (Arnol’di, 1977), *T*. *nadigi* (Kutter, 1925), *T*. *turcicus* (Santschi, 1934), ([16], Tamás Jégh pers. comm.).

### *Temnothorax stumperi* (Kutter, 1950)

*Epimyrma stumperi* Kutter [59]: 340, (m.) SWITZERLAND.

Combination in *Myrmoxenus*: Schulz & Sanetra [25]: 162.

Combination in *Temnothorax*: Ward et al. [29]: 75.

Examined type material.

One syntype gyne of *Epimyrma stumperi* was investigated from Switzerland: Flawil, VIII.1950 (1-ZML, CASENT0907577).

**Fig 28:**
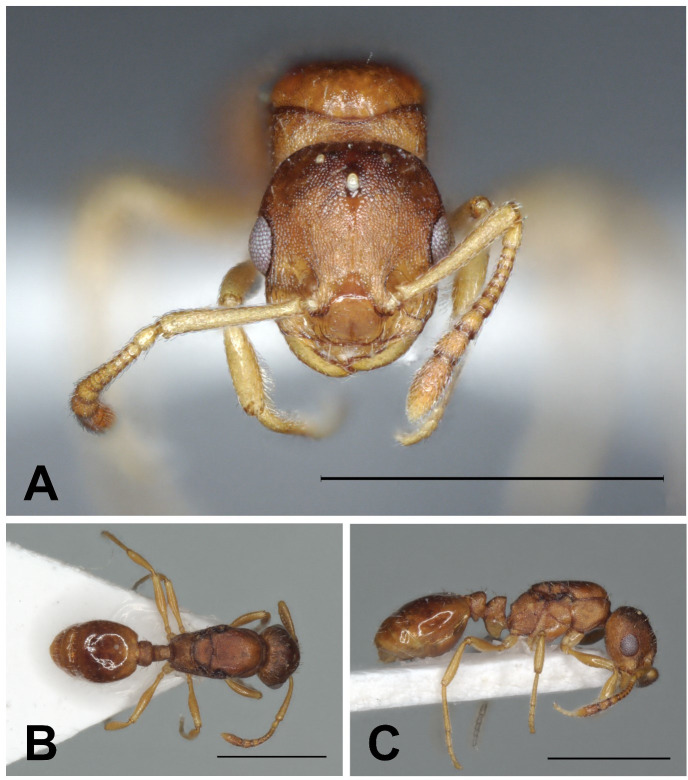
*Temnothorax stumperi* gyne. Head in full-face view (A), dorsal view of the body (B), lateral view of the body (C), scale bar: 1 mm.

Description of gynes (Fig 28 and Table 4).

Body color brown. Body color pattern concolorous.

Head.

Absolute cephalic size: 583±8.563 [569, 604]. Cephalic length vs. maximum width of head capsule (CL/CWb): 1.097±0.024 [1.061, 1.148]. Color: Brown. Frons: Brown. Vertex: Brown. Scape: Pale brown. Antennal club: Pale brown, brown. Head frontal sculpture: Reticulate with areolate ground sculpture. Clypeus: Smooth and shiny, with very few faint longitudinal ridges. Mandibles: Smooth, at long, t2-3 missing or very small, bt very small. Anterior margin of clypeus: Flat. Hairs: Sparse hairs erect allover except adpressed on the antennae. Hairs are thickening upwards, the ends chipped. Postocular distance vs. cephalic length (POC/CL): 0.363±0.009 [0.350, 0.382]. Eye vs. absolute cephalic size (EYE/CS): 0.291±0.008 [0.280, 0.312]. Frontal carina distance vs. absolute cephalic size (FR/CS): 0.343±0.012 [0.320, 0.359]. Scape length vs. absolute cephalic size (SL/CL): 0.718±0.019 [0.677, 0.757].

Alitrunk.

Color and color pattern: Brown. Sculpture: Dorsal region of mesosoma reticulate. Lateral region of pronotum reticulate. Anepisternum reticulate. Katepisternum reticulate. Metapleuron reticulate. Propodeal spines: Very short, slighlty pointed, triangulate. Hairs: Erect short hairs, mainly on the dorsal side of pronotum, mesonotum. Hairs are thickening upwards, the ends chipped. Mesosoma length vs. absolute cephalic size (ML/CS): 1.444±0.166 [1.396, 1543]. Maximum mesosoma width vs. absolute cephalic size (MW/CS): 0.850±0.037 [0.795, 0.925]. Spine length vs. absolute cephalic size (SPST/CS): 0.263±0.006 [0.251, 0.277]. Minimum spine distance at its base vs. absolute cephalic size (SPBA/CS): NA. Maximum spine distance at its tip vs. absolute cephalic size (SPWI/CS): 0.316±0.013 [0.287, 0.342]. Apical spine distance vs. absolute cephalic size (SPTI/CS): 0.269±0.014 [0.237, 0.292].

Pedicel.

Color: Brown. Sculpture: Petiole and postpetiole nodes reticulate, the ventral parts smooth. Petiole node: In profile, dorsal face of petiole node curving backwards without angle, sometimes slightly pointed anteriorly. Petiole ventral lobe/Ventral projection (lamella): In profile, posteroventral part of petiole forms convex or sometimes partly straight (but never concave) line. Ventral postpetiolar process/Inferior tooth: Slightly rounded. Hairs: Erect hairs on petiole, postpetiole nodes and ventral pp process. Petiole width vs. postpetiole width (PEW/PPW): 0.652±0.019 [0.596, 0.687].

Gaster.

Color: Brown. Hairs: Very short thin erect hairs on first tergite with a few longer hairs hickening upwards, the ends chipped. Sculpture: Smooth, shiny.

**Fig 29:**
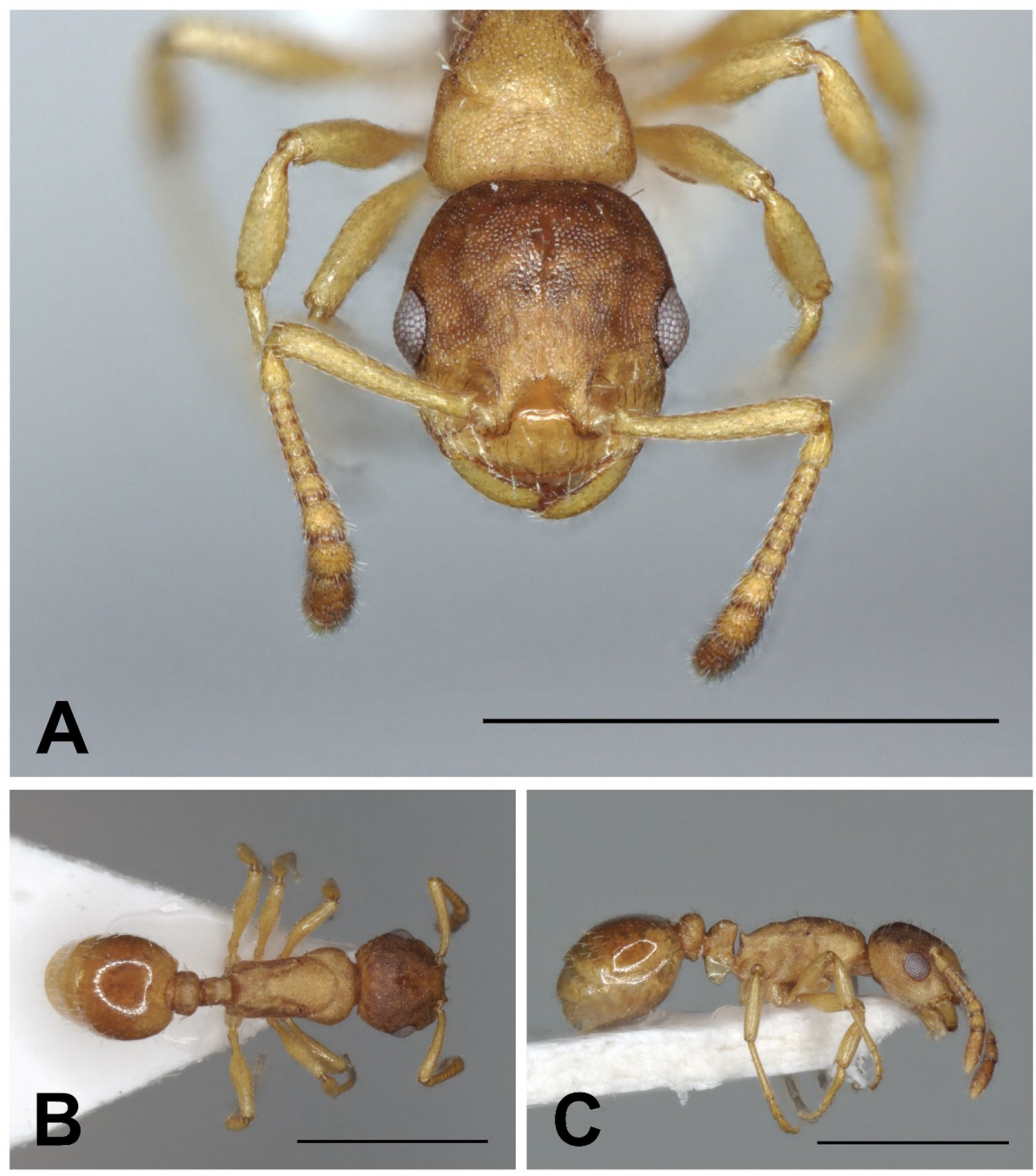
*Temnothorax stumperi* worker. Head in full-face view (A), dorsal view of the body (B), lateral view of the body (C), scale bar: 1 mm.

Description of workers (Fig 29 and Table 5).

Body color brown, pale brown. Body color pattern concolorous.

Head.

Absolute cephalic size: 582±16.108 [551, 618]. Cephalic length vs. maximum width of head capsule (CL/CWb): 1.143±0.020 [1.110, 1.188]. Color: Brown, pale brown. Frons: Brown, pale brown. Vertex: Brown. Scape: Pale brown. Antennal club: Brown. Head frontal sculpture: reticulate. Clypeus: Smooth, shiny with few longitudinal ridges. Mandibles: Smooth, shiny, 3–4 teeth, at long, t2-3 very short, rounded or reducated, bt short, rounded. Anterior margin of clypeus: Flat. Hairs: Short hairs erect allover except adpressed on the antennae. Hairs are thickening upwards, the ends chipped. Postocular distance vs. cephalic length (POC/CL): 0.358±0.013 [0.335, 0.395]. Eye vs. absolute cephalic size (EYE/CS): 0.253±0.010 [0.229, 0.275]. Frontal carina distance vs. absolute cephalic size (FR/CS): 0.336±0.012 [0.313, 0.364]. Scape length vs. absolute cephalic size (SL/CL): 0.703±0.030 [0.561, 0.747].

Alitrunk.

Color and color pattern: Pale brown. Sculpture: Dorsal region of mesosoma reticulate. Lateral region of pronotum reticulate. Mesopleuron reticulate. Metapleuron reticulate. Propodeal spines: Very short, pointed, triangulate. Hairs: Short erect hairs mainly on dorsal face of pronotum, mesonotum and propodeum. Hairs are thickening upwards, the ends chipped. Mesosoma length vs. absolute cephalic size (ML/CS): 1.273±0.022 [1.224, 1.315]. Maximum mesosoma width vs. absolute cephalic size (MW/CS): 0.706±0.017 [0.663, 0.735]. Spine length vs. absolute cephalic size (SPST/CS): 0.244±0.014 [0.219, 0.268]. Minimum spine distance at its base vs. absolute cephalic size (SPBA/CS): 0.314±0.015 [0.279, 0.345]. Maximum spine distance at its tip vs. absolute cephalic size (SPWI/CS): 0.332±0.021 [0.291, 0.389]. Apical spine distance vs. absolute cephalic size (SPTI/CS): 0.303±0.020 [0.261, 0.355].

Pedicel.

Color: Pale brown. Sculpture: Petiole and postpetiole punctuated, reticulate, the ventral parts smooth. Petiole node: In profile, dorsal face of petiole node curving backwards without angle. Petiole ventral lobe/Ventral projection (lamella): In profile, posteroventral part of petiole forms convex or sometimes partly straight line. Ventral postpetiolar process/Inferior tooth: Slightly rounded. Hairs: Short erect hairs on petiole, postpetiole nodes and very short hairs on ventral pp process. Hairs are thickening upwards, the ends chipped. Petiole width vs. Postpetiole width (PEW/PPW): 0.637±0.024 [0.588, 0.716].

Gaster.

Color: Brown, pale brown. First tergite sometimes paler at base. Hairs: Erect hairs allover the gaster, shorter thin and longer erect hairs present. Hairs are thickening upwards, the ends chipped. Sculpture: Smooth, shiny.

Geographic distribution

It is a montane species, relatively isolated populations are found in the Alps (Austria, Northern Italy, and Switzerland) and the mountains of Türkiye (Fig 15). It is also known from Peloponnese (Greece) and Pyrenees (Spain) [46, 60].

Host ant usage

The host species was *Temnothorax nigriceps* in 11 of the 20 samples we examined, *T*. *tuberum* (Fabricius, 1775) in 2 samples, and no specimens of the host species were available for 7 samples. Other host species mentioned in the literature: *T*. *unifasciatus* ([31], Anne Freitag pers. comm.).

## Supporting information

S1 Table Details of the complete morphologically analyzed material. Sample codes, collection information, and depository of the investigated samples.
(XLSX)

S2 Table Host usage data of the investigates parasite species. Sample codes, host species and parasite species information related to investigates samples.
(XLS)
