# Correction: Taxonomy of the Palearctic socially parasitic *Temnothorax* (*Myrmoxenus*) ants (Hymenoptera: Formicidae)

**DOI:** 10.1371/journal.pone.0327682

**Published:** 2025-07-02

**Authors:** Ferenc Báthori, Bernhard Seifert, Jürgen Heinze, Kadri Kiran, Celal Karaman, Sándor Csősz

Figs 5 and 24 are incorrect. The background of the images should have been white. Additionally, there is an error in the caption for Fig 5. Please see the correct Figs 5 and 24 here, as well as the correct caption for Fig 5.

**Fig 5:**
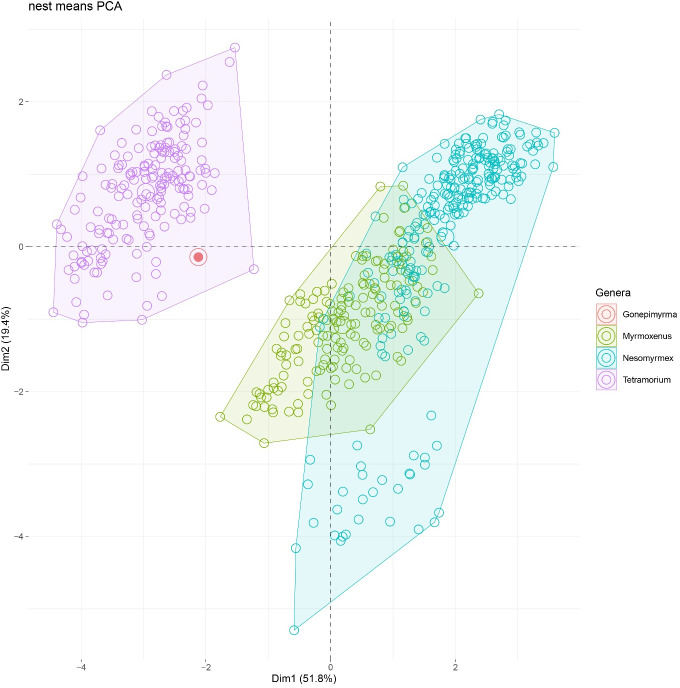
PCA plot of morphometric data of *Temnothorax corsicus* group species (green), *Nesomyrmex angulatus* group species (blue), and *Tetramorium* (lilac) nest samples and type material of *Tetramorium epimyrmoide* [formerly *Epimyrma* (*Gonepimyrma*) *africana* Bernard, 1948] (red) illustrated on two principal components (Dim 1, Dim 2).

**Fig 24:**
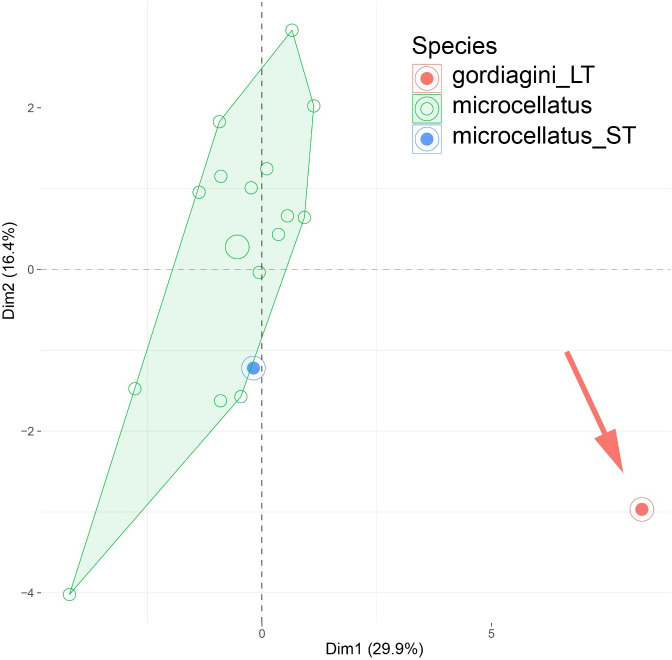
PCA plot of morphometric data of *Temnothorax microcellatus* (green) and the relevant type material are illustrated on two principal components (Dim 1, Dim 2). The syntype worker of *T. microcellatus* (blue) is nested in the *microcellatus* cluster and *T. gordiagini* (red) takes a peripheral position.
